# A naturally occurring standalone TrpB enzyme provides insights into allosteric communication within tryptophan synthase

**DOI:** 10.1002/pro.70103

**Published:** 2025-03-18

**Authors:** Thomas Kinateder, Lukas Drexler, Cristina Duran, Sílvia Osuna, Reinhard Sterner

**Affiliations:** ^1^ Institute of Biophysics and Physical Biochemistry, Regensburg Center for Biochemistry University of Regensburg Regensburg Germany; ^2^ Institut de Química Computacional i Catàlisi (IQCC) and Departament de Química Universitat de Girona Girona Spain; ^3^ ICREA Barcelona Spain

**Keywords:** allostery, molecular dynamics simulations, multi‐enzyme complexes, shortest path map, stand‐alone enzyme, tryptophan synthase

## Abstract

Allosteric regulation of catalytic activity is a widespread property of multi‐enzyme complexes. The tryptophan synthase is a prototypical allosteric enzyme where the constituting α (TrpA) and β (TrpB) subunits mutually activate each other in a manner that is incompletely understood. Experimental and computational studies have shown that LBCA‐TrpB from the last bacterial common ancestor contains six residues (Res_6_) distal from the active site that allow for high stand‐alone catalytic activity in the absence of a TrpA subunit. In the present study, a database search revealed that Res_6_ is also present in the extant *pl*TrpB from *Pelodictyon luteolum*. The *pl*TrpB enzyme showed a high stand‐alone activity and only a moderate activation by *pl*TrpA. The replacement of LBCA‐Res_6_ in *pl*TrpB with the consensus residues from a multiple sequence alignment yielded *pl*TrpB‐con, which showed a dramatically decreased stand‐alone activity but was strongly stimulated by *pl*TrpA. These findings suggest that the effect of these six key allosteric residues is largely independent of the protein context within a specific TrpB enzyme. Analysis of the conformational landscapes of *pl*TrpB and *pl*TrpB‐con revealed that *pl*TrpB in isolation displays efficient closure of both the active site and the communication (COMM) domain. In contrast, these catalytically competent states are destabilized in *pl*TrpB‐con but can be recovered by the addition of *pl*TrpA. A correlation‐based shortest path map (SPM) analysis reveals that the catalytically and allosterically relevant domains—specifically, the COMM domain in TrpB and loops 2 and 6 in TrpA—are tightly interconnected exclusively in *pl*TrpA:*pl*TrpB‐con.

## 1 INTRODUCTION

The term allostery describes the regulation of a biological macromolecule through the binding of a small effector molecule to a position distant from the functional site (Hofmann, 2023; Monod et al., 1963). Allostery is a widespread phenomenon that can be observed, for example, in the context of cell signaling and receptor proteins (Cournia & Chatzigoulas, 2020), oligomeric proteins like hemoglobin that cooperatively bind a ligand, or enzymes in which the catalytic activity is fine‐tuned by an inhibiting or an activating molecule (Lisi & Loria, 2017).

Comprehending allostery at a molecular level is key to unraveling central questions at the heart of molecular biology. In the context of biocatalysis, a thorough understanding and the ability to tune allostery will unlock the catalytic potential of metabolic enzymes, paving the way to a sustainable and green bioproduction of fine chemicals (Buller et al., 2015; Murciano‐Calles et al., 2016). The impact of mutations distal from the active site on the catalytic activity of enzymes indicates that a considerable amount of long‐range allosteric effects takes place in many enzymatic systems (Gunasekaran et al., 2004; Osuna, 2021). Several models were put forward to explain allostery, assuming either the propagation of an input signal via interconnecting residues (allosteric network model) or an equilibrium between states of low and high activities (conformational selection and population shift model) (Liu & Nussinov, 2016). Despite considerable efforts, the mechanistic basis of allostery, in general, is still poorly understood (Hofmann, 2023), and additional studies are needed to further our understanding of this widespread molecular feature.

A popular model enzyme to study allostery is tryptophan synthase (TS) because it combines two enzymatic activities that tightly allosterically regulate each other to synchronize the overall reaction (Brzović et al., 1992; Dunn, 2012; Kirschner et al., 1991). In bacteria, TS occurs as αββα heterotetramer consisting of two different subunits, α (TrpA) and β (TrpB), whereby the αβ (TrpA:TrpB) dimer forms the functional unit that is responsible for the last two steps in tryptophan biosynthesis (Goldberg et al., 1966; Yanofsky & Crawford, 1972). Specifically, TrpA catalyzes the retro‐aldol reaction of indole‐3‐glycerol‐phosphate (IGP) to glyceraldehyde‐3‐phosphate (G‐3‐P) and indole (Lane & Kirschner, 1991; Yanofsky & Crawford, 1972), which is released into a hydrophobic intermolecular channel that connects the active site of TrpA to the active site of TrpB (Dunn et al., 1990; Hyde et al., 1988). Meanwhile, in the TrpB active site, serine reacts with the cofactor pyridoxal phosphate (PLP) to form an aminoacrylate (AA). Then, AA reacts with indole to form tryptophan while the catalytic cofactor PLP is recovered (Figure S1) (Raboni et al., 2009). To prevent futile reactions catalyzed either by TrpA or TrpB, several layers of allosteric regulation have evolved. First, both TrpA and TrpB are generally activated upon complex formation (Hatanaka et al., 1962; Yanofsky & Crawford, 1972). Second, the binding of IGP to the TrpA active site results in a lowered Michaelis constant for serine (K_M_^Ser^) in TrpB (Dunn, 2012; Dunn et al., 1990), and third, the formation of the AA intermediate in TrpB leads to the catalytically activated conformation of TrpA, which enhances its turnover number (*k*_cat_) for the TrpA reaction (Anderson et al., 1991; Banik et al., 1995).

While the current models are still insufficient to explain all aspects of the allosteric communication within the TS at a molecular level, it is known that the so‐called COMM domain, which covers the active site in TrpB, plays an important role in allosteric signaling (Miles, 1991). Moreover, an (extended) open conformation of the COMM domain has been attributed to an inactive state of the TrpB monomer, whereas a closed conformation is associated with the catalytically competent state of TrpB in complex with TrpA (Brzović et al., 1992; Buller et al., 2018; Dunn et al., 2008; Maria‐Solano et al., 2019).

**FIGURE 1:**
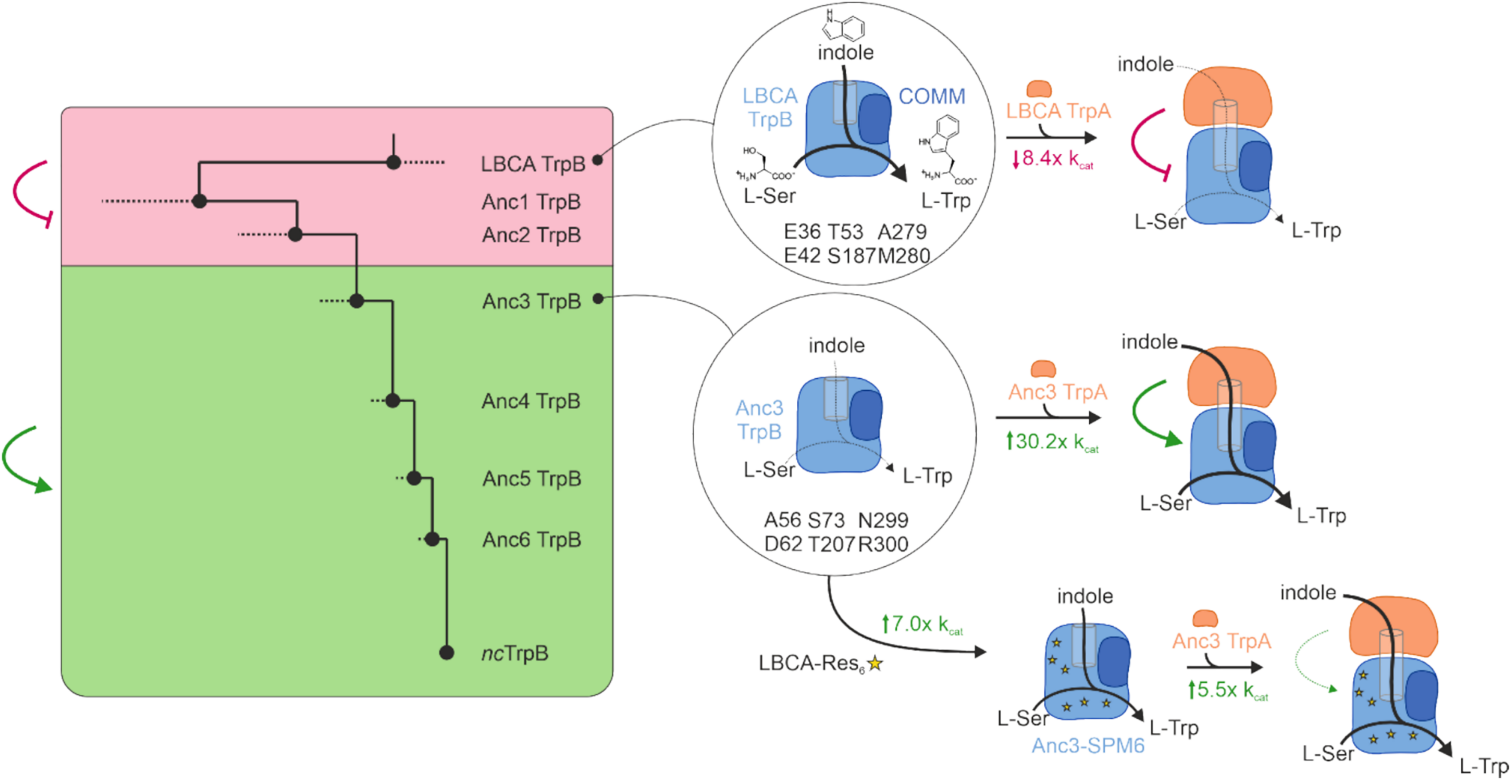
Changes in the stand‐alone activity of TrpB during evolution and based on computational predictions. Left panel: Ancestral sequence reconstruction of TrpB identified a switch from stand‐alone TrpB variants that were highly active on their own but were deactivated upon complex formation with TrpA (highlighted in red) to variants that were poorly active on their own but were activated upon complex formation with TrpA (highlighted in green) (Schupfner et al., 2020). Upper right panel: The shortest path map (SPM) method previously identified six residues Res_6_ in LBCA (E36, E42, T53, S187, A279, M280), which were assumed to be responsible for the 8.4‐fold inactivation by TrpA (Maria‐Solano et al., 2021). Middle right panel: The corresponding residues Res_6_ in Anc3 TrpB (A56, D62, S73, T207, N299, R300) result in a 30.2‐fold activation by TrpA. Lower right panel: The replacement of Res_6_ in Anc3 TrpB by Res_6_ from LBCA (LBCA‐Res_6_) led to Anc3‐SPM6, which shows a 7.0‐fold improved stand‐alone activity and only a 5.5‐fold activation by TrpA (Maria‐Solano et al., 2021).

In the past, several studies reported on TrpB variants that were freed from their regulation by TrpA, including work using directed evolution (Buller et al., 2015; Murciano‐Calles et al., 2016). In our own work, which was based on ancestral sequence reconstruction (ASR) (Schupfner et al., 2020), a remarkable functional switch was observed from the last common ancestor of all bacterial TrpB enzymes (LBCA TrpB) to an extant TrpB variant. Variants that were reasonably active on their own and were deactivated upon TrpA binding, that is, “stand‐alone” TrpBs, were substituted along the evolutionary trajectory by TrpB variants that were poorly active on their own and were strongly activated by TrpA binding (Figure 1) (Schupfner et al., 2020). Subsequently, we applied a correlation‐based computational method called shortest path map (SPM (Casadevall et al., 2024; Osuna, 2021)) to Anc3TrpB, that is, the first ancestral TrpB presenting allosteric activation, for enhancing its stand‐alone activity, thus freeing it from dependence on TrpA activation. SPM identifies those residues within a protein that show correlated motions in Molecular Dynamics (MD) simulations and hence are thought to define the conformational equilibrium between different functional states of the enzyme (Casadevall et al., 2024; Maria‐Solano et al., 2021; Osuna, 2021). The SPM conformationally relevant positions, together with sequence comparison between Anc3 and LBCA TrpB and thorough experimental characterization, identified six crucial residue positions (Res_6_), most of which are located distal from the active site (Figure S2). The identity of these six residues accounted for most of the observed functional changes between the stand‐alone TrpBs and the allosterically controlled TrpBs, which is exemplarily shown for LBCA TrpB and Anc3 TrpB in Figure 1. Importantly, when we introduced the subset of these LBCA‐Res_6_ from LBCA TrpB into the context of Anc3 TrpB, we observed an increase in the stand‐alone activity (*k*_cat_) by a factor of 7.0 and a reduction of the allosteric activation exerted by TrpA from 30.2‐fold to only 5.5‐fold (Figure 1).

Having identified the LBCA‐Res_6_, we wondered whether the functional effect of this set of residues was dependent on the TrpB context. Therefore, we searched for extant TrpB sequences containing LBCA‐Res_6_ and investigated whether they also show high stand‐alone activity and little dependence on their complementing TrpA partner. Our combined experimental and computational analysis indicates that the effect of these SPM‐ASR mutations might be context‐independent, as they exert a profound effect on the conformational landscape at different key reaction intermediates, thus impacting the long‐range allosteric interaction between TrpA and TrpB subunits.

## 2 RESULTS

In previous work, we identified six TrpB residues from LBCA, which are E36, E42 located at the surface, T53 at the β‐β interface, S187 at the active site, and A279, M280 at the α‐β interface (Figure S1). The introduction of these LBCA‐Res_6_ into Anc3 TrpB (A56E + D62E + S73T + T207S + N299A + R300M) led to an increase in its stand‐alone activity by 7.0‐fold in terms of *k*_cat_ and a reduction of its allosteric activation by TrpA from 30.2‐fold to 5.5‐fold (Figure 1). To check whether LBCA‐Res_6_ were retained in any extant TrpB variant, we retrieved all 6373 sequences annotated as TrpB from the KEGG database (as of December 2021) and constructed an MSA. By filtering the dataset for the occurrence of one of these 6 amino acids at a time, we identified TrpB from *Pelodictyon luteolum* (*pl*TrpB) as the only sequence that possesses the whole Res_6_ set.

To test the stand‐alone activity of *pl*TrpB and its allosteric regulation by *pl*TrpA, the corresponding genes were codon‐optimized for *Escherichia coli*, cloned into an appropriate vector, and expressed. Following the purification of recombinant *pl*TrpB and *pl*TrpA by affinity chromatography and size‐exclusion chromatography, the purity of the enzymes was confirmed by SDS‐PAGE (Figure S3) and their structural integrity was validated by far‐UV CD spectroscopy (Figure S4).

Steady‐state kinetic experiments were performed in which TrpB activity was assessed by a spectrophotometric assay directly observing the changes in absorption between indole and l‐Trp. In the absence of *pl*TrpA, *pl*TrpB showed a *k*_cat_ value of 0.35 s^−1^ and a catalytic efficiency (*k*_cat_/*K*_M_) of 17 M^−1^ s^−1^ for serine and 8970 M^−1^ s^−1^ for indole (Figure S5 and Table 1). While both *k*_cat_ and *k*_cat_/*K*_M_^Ind^ were comparatively high for an isolated TrpB enzyme, *k*_cat_/*K*_M_^Ser^ was surprisingly low, due to a remarkably high *K*_M_^Ser^. This prompted the question of whether l‐Ser was the native substrate of *pl*TrpB or, as in the case of TrpB2 enzymes, O‐phospho‐l‐serine (Busch et al., 2014). To test this, we used an HPLC‐based assay to investigate *pl*TrpB activity with O‐phospho‐l‐serine and other possible substrates (d‐serine, O‐acetyl‐l‐serine, O‐phospho‐d‐serine, O‐phospho‐l‐threonine, l‐threonine, and l‐cysteine). However, only in the presence of l‐serine complete turnover of indole to l‐tryptophan was detected under the given experimental conditions (Figure S6).

**TABLE 1:** Steady‐state enzyme kinetic parameters at 30°C of *pl*TrpB and *pl*TrpB‐con in isolation and in complex with *pl*TrpA.

	*k*_cat_ [s^−1^]	*K*_M_^Ser^ [mM]	*K*_M_^Ind^ [μM]	*k*_cat_/*K*_M_^Ser^ [s^−1^ M^−1^]	*k*_cat_/*K*_M_^Ind^ [s^−1^ M^−1^]
*pl*TrpB	0.35 ± 0.02	21 ± 3	39 ± 7	17 ± 2.6	8970 ± 1720
*pl*TrpA:*pl*TrpB	0.93 ± 0.21	5.6 ± 0.4	65 ± 7	166 ± 39	14,300 ± 3510
*pl*TrpB‐con	0.009 ± 0.002	5.8 ± 0.7	59 ± 3	1.6 ± 0.39	153 ± 34
*pl*TrpA:*pl*TrpB‐con	0.21 ± 0.06	0.013 ± 0.001	380 ± 76	16,200 ± 4770	553 ± 193

The shown data are the average and standard deviation of values obtained by triplicate measurements. Experimental conditions included 50 mM potassium phosphate (pH 7.5), 180 mM KCl, 40 μM PLP, saturating concentrations of one substrate (indole/l‐serine) and varying concentrations of the other substrate (l‐serine/indole). The individual substrate saturation curves and the values determined from their fitting to the Michaelis–Menten equation are shown in Figure S5.

**FIGURE 2:**
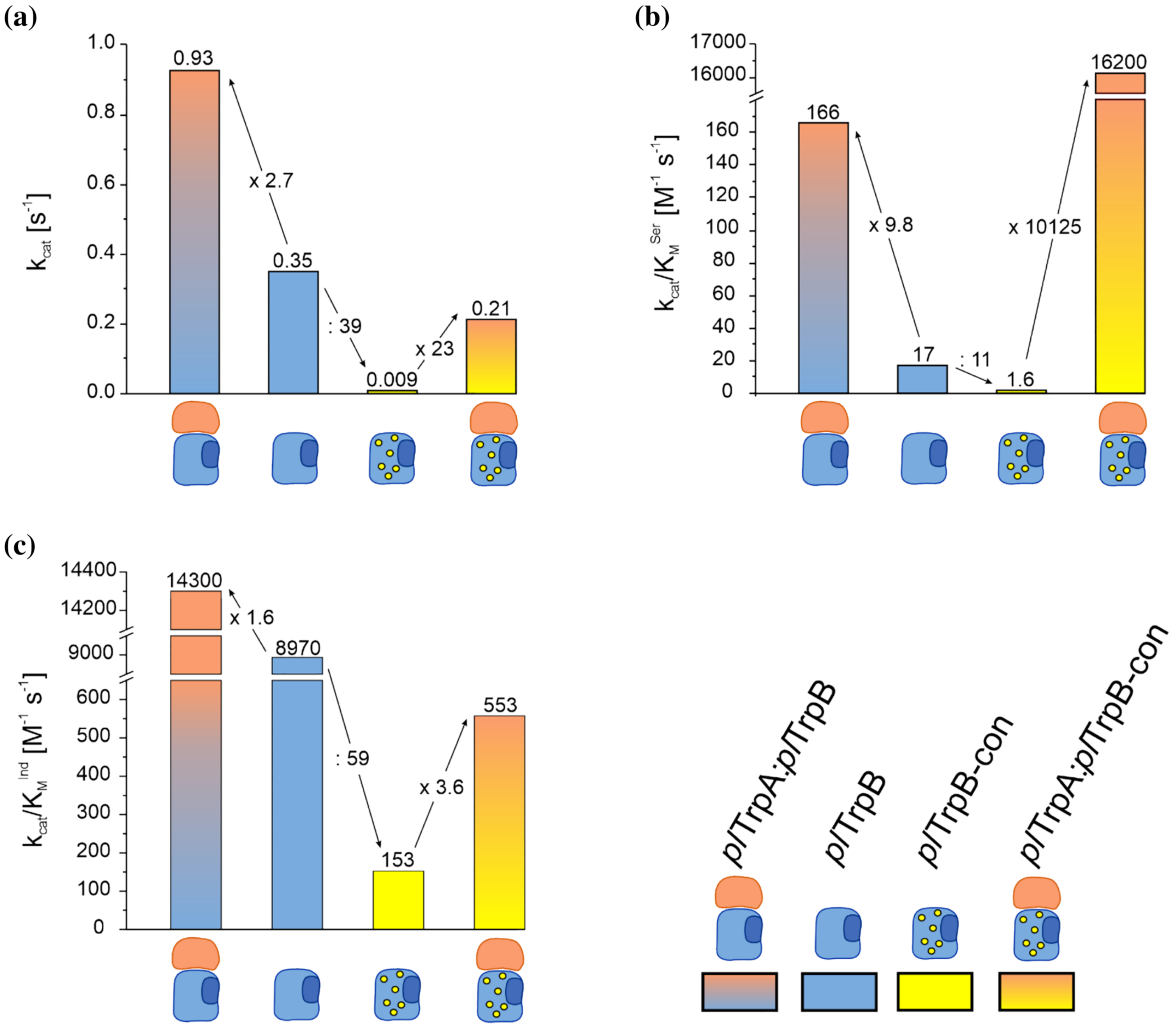
Steady‐state kinetic parameters *k*_cat_ (a), *k*_cat_/*K*_M_^Ser^ (b), and *k*_cat_/*K*_M_^Ind^ (c) of *pl*TrpB and *pl*TrpB‐con in isolation and in complex with *pl*TrpA. The data are taken from Table 1 and present the fold change in catalytic parameters resulting from either the addition of *pl*TrpA (indicated in orange) or the mutation of the Res_6_ to the consensus (indicated as yellow spheres).

Next, we investigated the effect of complex formation with *pl*TrpA on the catalytic activity of *pl*TrpB. In the presence of a 1.5‐fold molar excess of *pl*TrpA over *pl*TrpB, we recorded a *k*_cat_ of 0.93 s^−1^, and catalytic efficiencies of 166 M^−1^ s^−1^ for serine and 14,300 M^−1^ s^−1^ for indole (Figure S5 and Table 1). This translates to a moderate activation with respect to *k*_cat_ by a factor of 2.7 (Figure 2a), for *k*_cat_/K_M_^Ser^ by a factor of 9.8 (Figure 2b), and for *k*_cat_/*K*_M_^Ind^ by a factor of 1.6 (Figure 2c). Taken together, these results suggest that—except for the low apparent affinity to l‐serine—*pl*TrpB exploits a substantial fraction of its catalytic potential already in the absence of *pl*TrpA, supporting that the LBCA‐Res_6_ conveys stand‐alone activity independent of the protein context.

To further substantiate this hypothesis, we exchanged the LBCA‐Res_6_ of *pl*TrpB with the consensus amino acid found at each position as determined from the MSA of all TrpB1 enzymes. The resulting *pl*TrpB‐con variant contained the substitutions E46A+E52D+T63S+S197T+A289G+M290N. *pl*TrpB‐con was produced in the same way as wild‐type *pl*TrpB, and its purity and structural integrity were again experimentally confirmed by means of SDS‐PAGE and CD spectroscopy (Figures S3 and S4).

We next performed steady‐state kinetic experiments with *pl*TrpB‐con in isolation, which yielded a *k*_cat_ of 0.009 s^−1^ and catalytic efficiencies of 1.6 M^−1^ s^−1^ for serine and 153 M^−1^ s^−1^ for indole (Figure S5 and Table 1). Compared to *pl*TrpB, these values correspond to a large decrease of *k*_cat_ by a factor of 39 (Figure 2a), of *k*_cat_/*K*_M_^Ser^ by a factor of 11 (Figure 2b), and of *k*_cat_/*K*_M_^Ind^ by a factor of 59 (Figure 2c). Obviously, these reductions could be either caused by a significantly reduced catalytic potential of *pl*TrpB‐con compared to *pl*TrpB or a much stronger dependency of *pl*TrpB‐con on allosteric activation by *pl*TrpA. To discriminate between these two alternatives, we measured the activity of *pl*TrpB in the presence of a 1.5‐fold excess of *pl*TrpA. Remarkably, the determined *k*_cat_ value was 0.21 s^−1^, while the catalytic efficiencies were 16,200 M^−1^ s^−1^ for serine and 553 M^−1^ s^−1^ for indole, which corresponds to activations of *k*_cat_ by a factor of 23 (Figure 2a), of *k*_cat_/*K*_M_^Ser^ by a factor of more than 10,000 (Figure 2b), and of *k*_cat_/*K*_M_^Ind^ by a factor of 3.6 (Figure 2c). Interestingly, the *k*_cat_/*K*_M_^Ser^ of the *pl*TrpA:*pl*TrpB‐con complex surpassed the value for the *pl*TrpA:*pl*TrpB complex by approximately two orders of magnitude.

To exclude that the observed increased *pl*TrpA‐induced allosteric activation of *pl*TrpB‐con compared to *pl*TrpB is caused by higher subunit binding affinities in the *pl*TrpA:*pl*TrpB‐con than in the *pl*TrpA:*pl*TrpB complex, we determined apparent *K*_d_^app^ values by activity titrations. For this, we added varying concentrations of *pl*TrpA to a constant concentration of *pl*TrpB or *pl*TrpB‐con and measured the reaction rate at saturating substrate concentrations. The determined apparent dissociation constants *K*_d_^app^ of 8.5 μM for *pl*TrpA:*pl*TrpB (Figure S7) and 9.9 μM for *pl*TrpA:*pl*TrpB‐con (Figure S8) indicate that the fractions of *pl*TrpB and *pl*TrpB‐con complexed by *pl*TrpA as present in our steady‐state kinetic measurements were basically identical. This finding was confirmed by analytical size exclusion chromatography. The determined elution volumes showed that *pl*TrpA in isolation forms α‐monomers, whereas for both *pl*TrpB and *pl*TrpB‐con a ββ‐dimer was detected. Mixing of *pl*TrpA with *pl*TrpB or *pl*TrpB‐con yielded similar fractions of isolated α, isolated ββ, as well as αββ and αββα complexes for *pl*TrpA:*pl*TrpB and *pl*TrpA:*pl*TrpB‐con, respectively (Figure S9). To estimate the theoretical allosteric activation for complete αββα complex formation, we performed a simple calculation based on the law of mass action, according to which the actual *k*_cat_ values of *pl*TrpB and *plTrpB*‐con in the respective complexes may have been underestimated by factors of 2.5 for *pl*TrpA:*pl*TrpB and 3.6 for *pl*TrpA:*pl*TrpB‐con, respectively (Calculation S1). Hence, the allosteric activation by *pl*TrpA is even more pronounced for *pl*TrpB‐con compared to *pl*TrpB than has been estimated from the results of the steady‐state measurements listed in Table 1.

**FIGURE 3:**
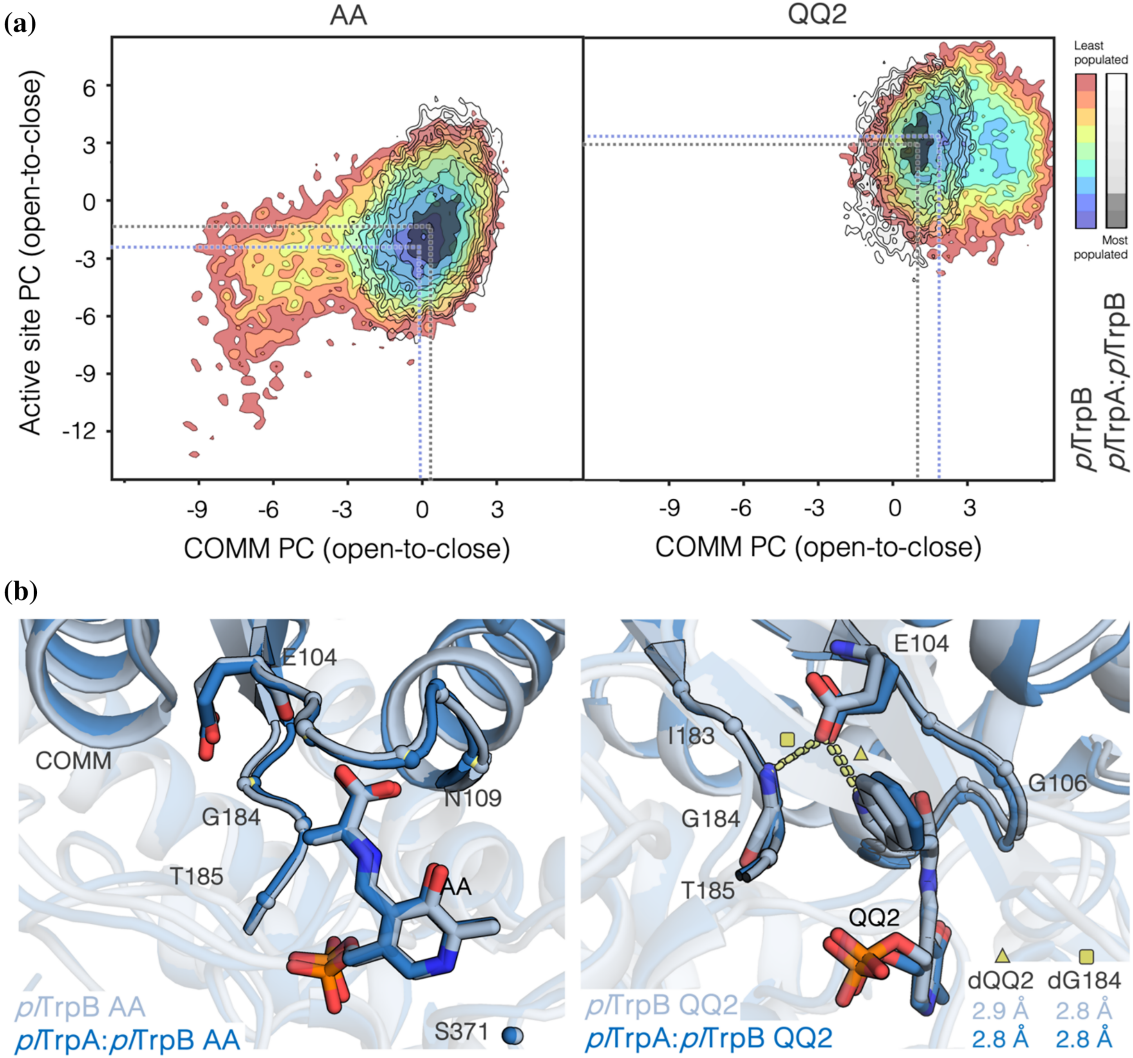
Reconstructed conformational landscapes of *pl*TrpB in isolation and in the *pl*TrpA:*Pl*TrpB complex at the aminoacrylate AA (left) and quinonoid QQ2 (right) reaction intermediates. (a) Overlay of the PCA‐generated conformational landscape considering distances between Cα‐atoms of the residues included in the COMM domain (x‐axis) and active site (y‐axis). Negative values of PC correspond to open states, whereas positive values correspond to closed states. The conformational landscape of *pl*TrpB is represented using a red‐to‐blue coloring scheme (red for the least populated conformation and blue for the most populated ones), whereas the landscape of the *pl*TrpA:*Pl*TrpB complex is shown with black lines (the most stable minimum is colored in black). (b) Overlay of two representative structures extracted from the most stable minima for *pl*TrpB (light blue) and *pl*TrpA:*Pl*TrpB (dark blue) at the AA (left panel) and QQ2 (right) intermediates. The mean distance between E104 and G184 or indole nitrogen at the QQ2 state is represented in Å.

We next asked why *pl*TrpB is much more active than *pl*TrpB‐con and why *pl*TrpB‐con is much more efficiently allosterically activated by *pl*TrpA, even though most of the differences between the two proteins are conservative amino acid exchanges. To address these questions, we resorted to a computational analysis of the two *pl*TrpB variants in isolation and in complex with *pl*TrpA, considering two key reaction intermediates: the aminoacrylate (AA) Schiff base and the quinonoid II (QQ2) (Figure S1). In a previous study, we found that the evaluation of the conformational landscape of *pf*TrpB from *Pyrococcus furiosus* and some stand‐alone variants at these two reaction intermediates was crucial for rationalizing the changes in stand‐alone activity and allosteric regulation (Maria‐Solano et al., 2019). Since the catalytic proficiency of TrpB depends on efficient closure of the active site and allosteric communication mediated by the COMM domain (Dunn et al., 2008), we decided to reconstruct the conformational landscapes based on Principal Component analysis (PCA), focusing on these two parameters in our analysis (Section 4, Figure 3 and Figure S10).

The conformational landscape of *pl*TrpB alone and in the *pl*TrpA:*pl*TrpB complex reveals a higher flexibility of the COMM domain (*x*‐axis) in the case of *pl*TrpB, especially at the AA intermediate (Figure 3a), thus indicating that *pl*TrpB in the absence of *pl*TrpA can adopt both open and closed conformations. This can be interpreted as a restriction of the conformational space that is accessible for *pl*TrpB exerted by its binding partner *pl*TrpA. However, only minor differences are observed in the most populated conformation, as both *pl*TrpB and *pl*TrpA:*pl*TrpB share the same minima at closed conformations of the COMM and active site (minima are at ca. 0, −2 for AA and at ca. 1.5, 3 at QQ2, Figure 3a). The overlay of a representative structure of the most stable conformation for *pl*TrpB and *pl*TrpA:*pl*TrpB indeed shows minor structural differences at both the AA and QQ2 intermediates (Figure 3b), hence indicating that *pl*TrpB in the absence of *pl*TrpA can adopt the catalytically productive closed states of the COMM domain and active site. The small conformational deviations observed when comparing isolated *pl*TrpB with *pl*TrpA:*pl*TrpB are in line with the high stand‐alone activity (*k*_cat_) of *pl*TrpB (Table 1). In the case of the *pl*TrpA:*pl*TrpB complex, the COMM domain flexibility is restricted as the minimum appears to be overall more narrow at both the AA and QQ2 intermediates, which could explain the slight increase in the *k*_cat_ value upon complex formation. Furthermore, taking into account that *pl*TrpB and *pl*TrpA:*pl*TrpB represent the two best catalysts with respect to *k*_cat_, the shared minimum found in the two conformational landscapes presenting the COMM domain closed with a proper positioning of E104 to stabilize the positive charge of the nitrogen of the indole at QQ2 represents the catalytically productive conformation, which ideally stabilizes the transition state and leads to the highest turnover numbers.

Although in our MD simulations l‐Ser is already coupled to pyridoxal phosphate (AA intermediate), the higher conformational flexibility of the COMM domain observed in the case of *pl*TrpB, as well as a more open active site tunnel (Figure S11) might explain the high Michaelis constant *K*_M_^Ser^ observed experimentally (Table 1).

**FIGURE 4:**
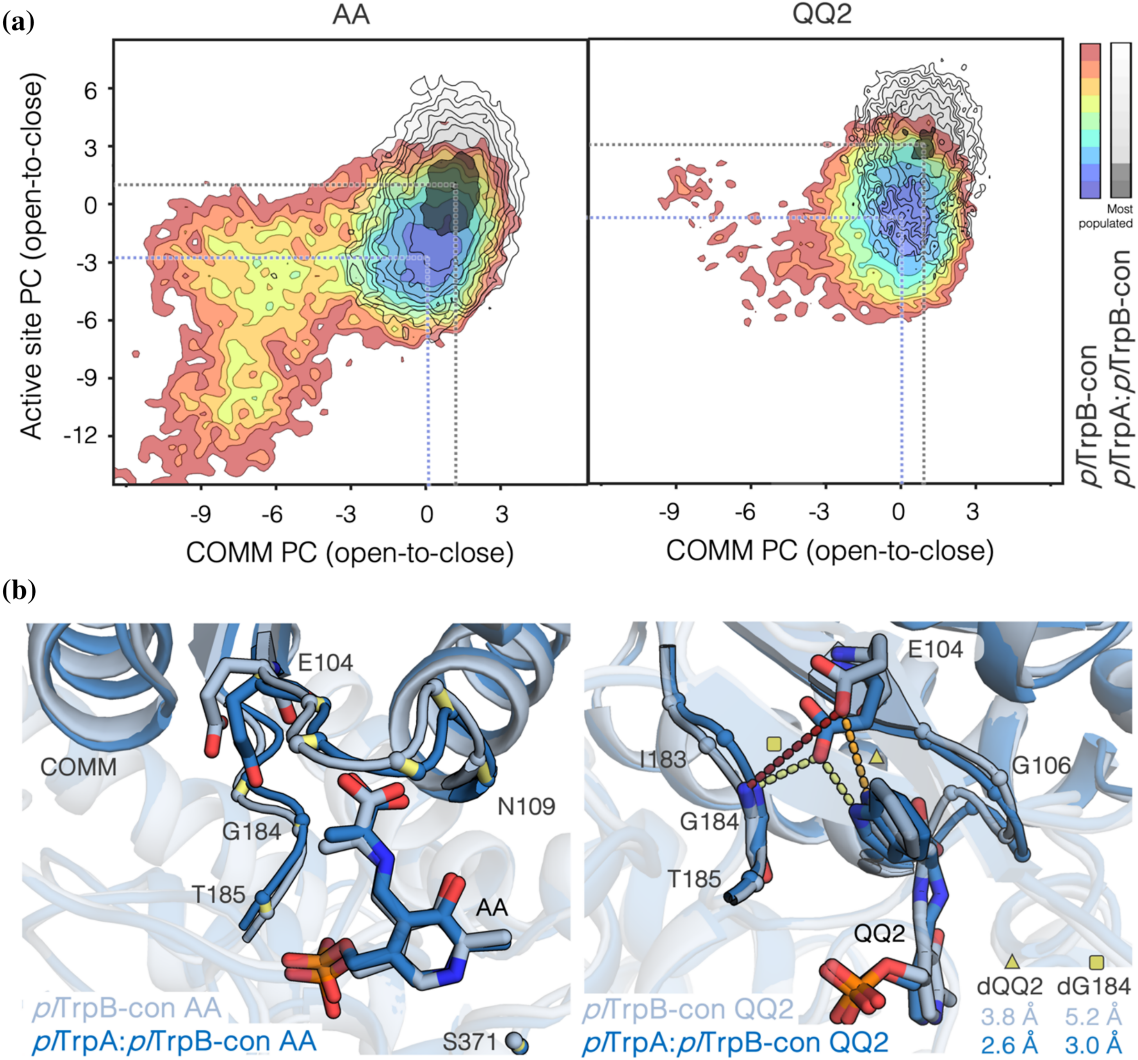
Reconstructed conformational landscapes of isolated *pl*TrpB‐con and complexed *pl*TrpB‐con in *pl*TrpA:*pl*TrpB‐con at the aminoacrylate AA (left) and quinonoid QQ2 (right) reaction intermediates. (a) Overlay of the PCA‐generated conformational landscape considering distances between Cα‐atoms of the residues included in the COMM domain (*x*‐axis) and active site (*y*‐axis). Negative values of PC correspond to open states, whereas positive ones correspond to closed states. The conformational landscape of *pl*TrpB‐con is represented using a red‐to‐blue coloring scheme (red for the least populated conformation, blue for the most populated ones), whereas the landscape of the *pl*TrpA:*pl*TrpB‐con complex is shown with black lines (the most stable minimum is colored in black). (b) Overlay of two representative structures extracted from the most stable minima for *pl*TrpB‐con (light blue) and *pl*TrpA:*pl*TrpB‐con (dark blue) at the AA (left panel) and QQ2 (right) intermediates. The mean distance between E104 and G184 or indole nitrogen at the QQ2 state is represented in Å.

Next, we analyzed the conformational landscapes of *pl*TrpB‐con in isolation and in the *pl*TrpA:*pl*TrpB‐con complex. Intriguingly, large differences are observed between *pl*TrpB‐con and *pl*TrpA:*pl*TrpB‐con: in the absence of the binding partner, *pl*TrpB‐con exhibits a high conformational heterogeneity for both the active site and COMM domain and, more importantly, the most stable conformation is substantially shifted when in complex with *pl*TrpA (Figure 4a). Specifically, the active site and the COMM domain exhibit a slightly more open conformation at both the AA and QQ2 intermediates in the case of *pl*TrpB‐con. The overlay of a representative structure of the most stable minima of *pl*TrpB‐con and *pl*TrpA:*pl*TrpB‐con (Figure 4b) indeed indicates suboptimal stabilization of QQ2 in *pl*TrpB‐con (i.e., a larger distance between E104 and the nitrogen of indole, Figure S12), which is in line with the poor *k*_cat_ value found for this variant. Some deviations of the COMM domain loop containing E104, G106, and the 183–185 loop are observed between isolated *pl*TrpB‐con and *pl*TrpB‐con in complex (Figure 4b). Interestingly, the conformational landscape of the *pl*TrpA:*pl*TrpB‐con complex resembles the one found for *pl*TrpA:*pl*TrpB; however, a shift in the location of the minimum towards a more closed active site conformation is observed for the former, especially at the AA intermediate. This means that *pl*TrpA seems to alter the conformational equilibrium, pushing it towards a more closed active site, which improves the Michaelis constant *K*_M_ for l‐Ser, while hampering indole access, thus yielding a higher *K*_M_ for indole in *pl*TrpA:*pl*TrpB‐con (Table 1). The analysis of the tunnels with respect to the COMM domain closure also evidences a narrower (more closed) tunnel in the case of *pl*TrpA:*pl*TrpB‐con, again explaining the elevated *K*_M_ for indole found experimentally (Table 1 and Figure S11). Our MD simulations therefore provide an explanation for the reversal of catalytic efficiency between indole and serine observed when comparing both *pl*TrpA:*pl*TrpB and *pl*TrpA:*pl*TrpB‐con complexes: The improved *K*_M_^Ser^ in *pl*TrpA:*pl*TrpB‐con is explained by the more closed TrpB active site pocket, which helps retain l‐Ser in a productive pose for the external aldimine 1 (Aex1) and AA formation. Such a closed conformation of the TrpB active site induces the closing of the TrpA‐TrpB tunnel, thus hampering indole diffusion and affecting its *K*_M_^Ind^. The opposite effect is observed in *pl*TrpA:*pl*TrpB, which presents a more open TrpB active site pocket detrimental for the productive coupling of l‐Ser to PLP, but also a more open TrpA–TrpB tunnel helping indole channeling and thus leading to a lower *K*_M_^Ind^ value compared to *pl*TrpA:*pl*TrpB‐con.

According to our computational and experimental results, standalone activity is obtained if the isolated subunit can efficiently stabilize the catalytically productive closed conformation of the COMM domain and active site, similar to what is observed in the presence of the allosteric binding partner. This is in line with our previous studies focused on comparing the allosterically driven conformational ensemble with that of isolated subunits of *pf*TrpB (Casadevall et al., 2022; Maria‐Solano et al., 2019). The kinetic characterization of isolated *pl*TrpB and *pl*TrpB‐con in the two corresponding complexes indicates that efficient allosteric activation is found if (i) the allosteric effector—in our case TrpA—restricts the conformational space of the allosterically controlled enzyme and (ii) catalytically productive states with closed conformations of the COMM domain and active site are stabilized over catalytically unproductive ones.

**FIGURE 5:**
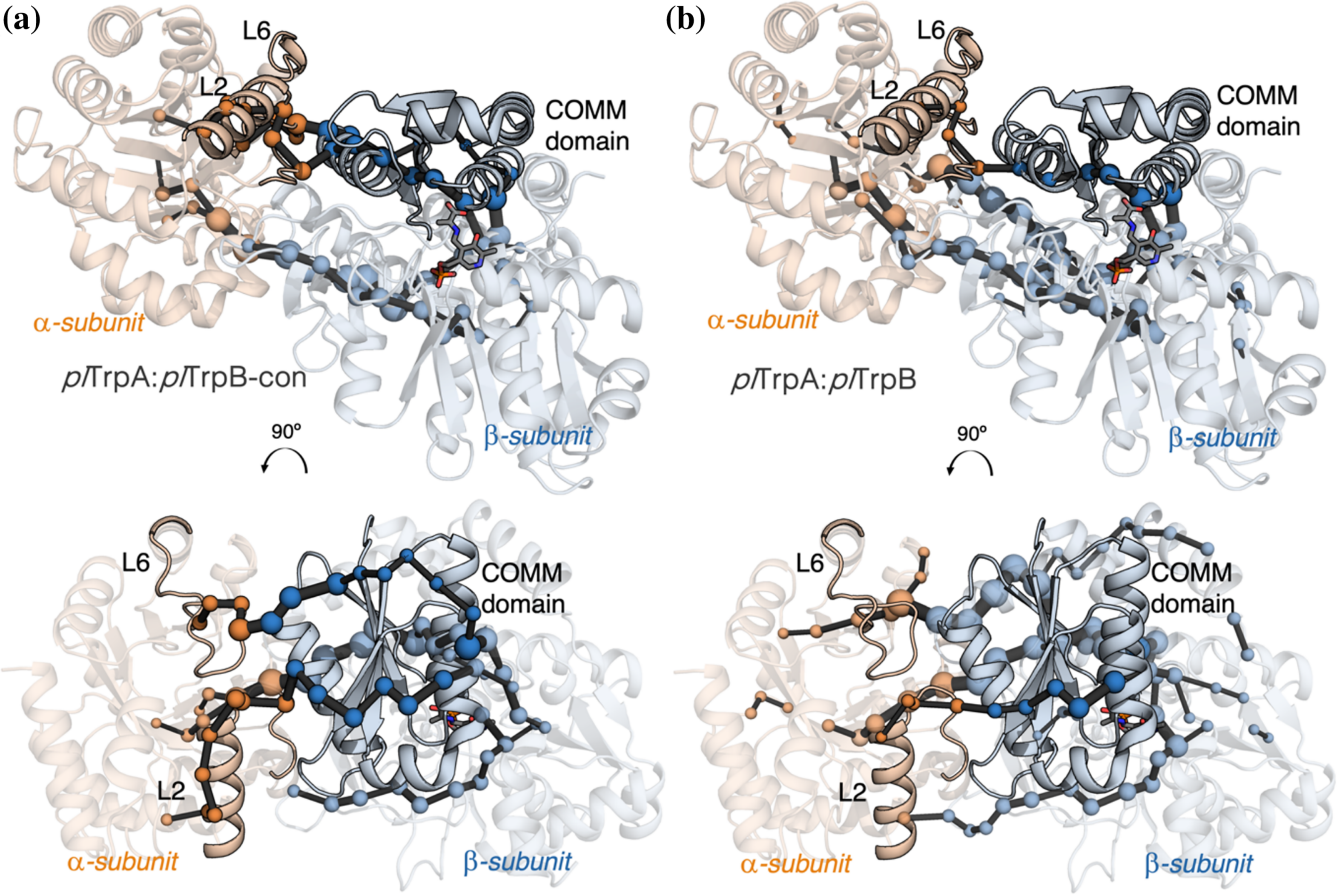
SPM‐identified allosteric communication pathway for *pl*TrpA:*pl*TrpB‐con (a) and *pl*TrpA:*pl*TrpB (b). The key structural elements described to be allosterically connected are labeled: loop 6 (L6), loop 2 (L2) in *pl*TrpA, and the COMM domain in *pl*TrpB/*pl*TrpB‐con. In both cases, the *pl*TrpA subunit is represented in orange, whereas *pl*TrpB/*pl*TrpB‐con is colored in blue.

This leads to the final question of how the mutations influence the communication between the two proteins. To address this problem, we evaluated the allosteric communication between *pl*TrpA and *pl*TrpB/*pl*TrpB‐con using our correlation‐based SPM tool and considering the AA intermediate in which the allosteric signal is hypothesized to be maximal (Dunn, 2012; Ito et al., 2022; Ito et al., 2023). The formation of the AA intermediate in TrpB stabilizes the catalytically activated closed conformation of TrpA in which both L6 and L2 cover the active site and promote the IGP retro‐aldol cleavage (Duran et al., 2024; Kulik et al., 2005). At the same time, the catalytically activated closed state of TrpA favors the closing of the COMM domain for retaining indole and promoting its coupling with AA. Interestingly, we observe such a sophisticated allosteric communication only in the case of *pl*TrpB‐con: the computed SPM shows an intertwined pathway connecting both active site loops 6 and 2 (L6 and L2) of *pl*TrpA with the COMM domain and the *pl*TrpB‐con active site where PLP is located (Figure 5a). In contrast, *pl*TrpB, which is substantially less activated by the presence of *pl*TrpA, preserves the communication between L2 and the COMM domain but lacks the direct connection with L6 (Figure 5b). Therefore, reverting LBCA‐Res_6_ to the consensus residues has led to a significant change in the dynamic properties and allosteric communication, which impacts activity and substrate affinity.

## 3 DISCUSSION

In comparison to previously studied extant TrpB enzymes, *pl*TrpB exhibits several unusual properties: a superior standalone activity, weak allosteric activation by *pl*TrpA, and poor binding affinity for its substrate l‐serine. The first two properties can be attributed to the presence of the LBCA‐Res_6_ residues (Figure 1), which obviously have persisted over time in this extant TrpB enzyme. Consequently, the same residues are responsible for a substantial enhancement of the stand‐alone properties of both an ancestral (LBCA TrpB) and an extant (*pl*TrpB) enzyme, and this effect is also observed in a previously computationally designed variant (Anc3TrpB‐SPM6) (Maria‐Solano et al., 2021). It is intriguing to note that this particular set of conformationally relevant residues seems to function independently of the specific protein context, as the comparison of the SPM networks between ancestral and multiple extant TrpBs from different organisms shows similar SPM pathways, all identifying these six positions as relevant (Figure S13). Of note is that this Res_6_ subset differs from previously identified sets of residues as deduced from MSAs that can promote standalone activity (Buller et al., 2015; Schupfner et al., 2020). Hence, there exist different sets of activating residues, which could be transferable to other TrpB enzymes, providing a potential strategy for enhancing their activity. In fact, in a recent publication, more than 500,000 variants of TrpB from *Thermotoga maritima* were reported using a continuous evolution strategy, in which key mutations at the enzyme surface, but also at the α–β interface, were found to be important for improving the stand‐alone function of *Tm*TrpB (Rix et al., 2024). Actually, one of these key positions was I271 (P271 in LBCA), which is located right next to two of the Res_6_ positions studied here, that is, A279 and M280 at the α–β interface.

These characteristics raise fundamental questions about the physiological roles of *pl*TrpB as well as *pl*TrpA within their host organism, *P. luteolum*. Based on the exclusive substrate specificity of *pl*TrpB for l‐serine, its sequence homology to other TrpB1 enzymes, and the presence of the characteristic catalytic Asp residue in the active site, this enzyme can be confidently classified as a member of the TrpB1 group (Table S1) (Fleming et al., 2018; Merkl, 2007; Xie et al., 2002). However, analysis of the genomic neighborhood of *pl*TrpB (Figure S14) reveals two striking differences compared to other TrpB1 enzymes: First, *pltrpA* and *pltrpB* are separated by nine interjacent genes. Second, both genes are encoded on different DNA strands and are thus not co‐transcribed. Also, neither *pltrpA* nor *pltrpB* is embedded in a *trp* operon. Based on the genomic proximity, further nucleophiles were tested as substrates that might replace indole; however, none of them showed any product formation (Figure S15a). As suggested by the stand‐alone properties of *pl*TrpB, the moderate affinity for *pl*TrpA, as shown by the relatively high *K*_d_^app^ value of 8.5 μM (Figure S7), and the isolated genomic location of *pltrpA* and *pltrpB*, the organism does not appear to be overly concerned with losing nascent indole from the *pl*TrpA:*pl*TrpB complex. One could speculate that *P. luteolum* possesses other pathways for indole biosynthesis and scavenges the metabolite by another enzyme. The existence of a TrpB2 protein, a class of TrpB homologues that have a high affinity for indole (Hettwer & Sterner, 2002) supports this hypothesis. Alternatively, uncoupling of *pl*TrpA and *pl*TrpB may allow the organism to utilize indole for processes other than tryptophan biosynthesis, suggesting that indole may serve as a precursor for other metabolites. Moreover, the stand‐alone nature of *pl*TrpB led us to hypothesize that it might play a role in l‐tryptophan degradation. We therefore analyzed the reverse reaction from l‐tryptophan to indole and l‐serine using an HPLC‐based enzyme assay. However, we found no evidence of indole production (Figure S15b). Further studies are needed to elucidate the precise role of this enzyme within the organism and to understand the underlying molecular mechanisms that account for its unique properties.

The observed decrease in both *k*_cat_ (39‐fold) and *k*_cat_/*K*_M_^Ser^ (11‐fold) when transitioning from wildtype –*pl*TrpB to *pl*TrpB‐con (Figure 2a,b) is striking. However, the subsequent dramatic increase upon the addition of *pl*TrpA (24‐fold increase for *k*_cat_, 10.125‐fold increase for *k*_cat_/*K*_M_^Ser^) is equally intriguing. Here, the introduction of mutations initially led to a significant decrease in enzymatic activity, which can, however, be restored upon the addition of an interaction partner. Moreover, in terms of *k*_cat_/*K*_M_,^Ser^ wildtype levels are even surpassed by two orders of magnitude. Our findings provide a conclusive example of sign epistasis, a phenomenon where mutations are detrimental or negative in one context but beneficial or positive in another context (Weinreich et al., 2005). This is particularly interesting from an evolutionary perspective because it means that mutations that may initially harm an enzyme can become beneficial through the acquisition of an interaction partner, resulting in a dependence on this partner. Such an effect has indeed been observed for the evolution of ribulose‐1,5‐bisphosphate carboxylase/oxygenase (Schulz et al., 2022). However, during the evolution of stand‐alone *pl*TrpB from stand‐alone LBCA TrpB, either a strong dependence on *pl*TrpA has never existed or the enzyme managed at some point during its evolution to free itself from this sign epistasis by acquiring residues that provided enhanced stand‐alone activity. The evaluation of the allosteric regulation pathway operating between subunits indeed indicates that the catalytically relevant COMM domain of *pl*TrpB is not optimally connected to the two active site loops of *pl*TrpA, thus explaining the low activation of *pl*TrpB in the presence of the allosteric binding partner *pl*TrpA. When analyzing the different enzyme variants and enzyme complexes computationally, we found a strong correlation between the activity of each variant and the shift and stabilization of the catalytically competent closed states at the reconstructed conformational landscapes. The shift that is observed in the energetic minima of *pl*TrpB‐con upon complex formation can be interpreted as the stabilization of the catalytically competent state of TrpB. In essence, our results can best be explained by the conformation selection and population shift model (Liu & Nussinov, 2016), as *pl*TrpA changes the distribution of different *pl*TrpB‐con conformations toward productive ones. This is also supported by the observation that the Res_6_ are scattered all over the protein and do not form a coherent network, which would be more in line with an allosteric network model.

## 4 MATERIALS AND METHODS

### 4.1 Bacterial strains and chemicals

All proteins that were analyzed in this study were expressed in the *E. coli* strain BL21 Gold (DE3), purchased from Agilent Technologies. All chemicals used in this study were purchased from commercial sources and were of analytical grade or higher.

### 4.2 Cloning

The genes for *pl*TrpA, *pl*TrpB, and *pl*TrpB‐con were purchased from GeneArt (Thermo Fisher Scientific). They were codon‐optimized for recombinant gene expression in *E*. *coli* and equipped with flanking *Bsa*I restriction sites. Then, the genes were cloned into pET21a_*Bsa*I expression vectors (Rohweder et al., 2018) using a coupled digestion/ligation reaction with *Bsa*I and T4‐DNA ligase. The resulting constructs allow for IPTG‐inducible expression with a C‐terminal His_6_‐tag. The deduced amino acid sequences of *pl*TrpA, *pl*TrpB, and *pl*TrpB‐con are shown in Table S2.

### 4.3 Gene expression and protein purification

The *E. coli* expression strain BL21 Gold (DE3) was transformed with the expression plasmids coding for *pl*TrpA, *pl*TrpB, and *pl*TrpB‐con, respectively. The cells were grown at 37°C in lysogenic broth (LB) medium supplemented with 150 mg/mL ampicillin to an OD_600_ of 0.6. Next, gene expression was induced by the addition of 0.5 mM IPTG, and the cultures were further incubated overnight at 20°C. Cells were then harvested by centrifugation (4000 g, 20 min) and resuspended in 50 mM KP (pH 7.5), 300 mM KCl, and 10 mM imidazole. Afterward, cells were disrupted by sonication (Branson Sonifier W‐250D, 60% amplitude, 2.5 min, 2 s pulse, 2 s pause) and cell debris and insoluble aggregates were removed by centrifugation (14,000 g, 45 min). All proteins analyzed in this study were expressed with a C‐terminal His_6_‐tag. The His_6_‐tagged proteins were purified from the supernatant by affinity chromatography using an ÄKTA‐purifier system with a HisTrap excel column (CV 5 mL, GE Healthcare) applying a linear imidazole gradient (10–500 mM over 15 CV). The proteins were further purified by preparative size exclusion chromatography using an ÄKTA‐purifier system with a Hi Load 16/600 Superdex 75 pg. column (CV 330 mL, GE Healthcare). The proteins were eluted in 50 mM potassium phosphate (pH 7.5) and 300 mM KCl. Fractions containing the purified protein were identified by SDS‐PAGE and pooled. In the case of *pl*TrpA, the protein concentration was determined by absorbance spectroscopy at 280 nm (Thermo Fisher Scientific, NanoDrop One) using a molar extinction coefficient of 26,930 (M^−1^ cm^−1^) (Wilkins et al., 1999). In the case of *pl*TrpB and *pl*TrpB‐con, due to the additional absorption of the PLP cofactor, the protein concentration was determined with the Bradford assay using a commercial Bradford reagent (Bio‐Rad, Bradford protein assay). The purified proteins were dripped into liquid nitrogen and stored at −70°C.

### 4.4 HPLC‐based substrate screening

An HPLC‐based substrate screening was performed to identify potential additional alpha‐amino acid substrates of *pl*TrpB that might be preferred as substrates over l‐serine. All enzymatic assays contained 500 μM indole, 100 mM potassium phosphate (pH 7.5), 180 mM KCl, 40 μM PLP, 2 mM of the respective alpha‐amino acid, and 5 μM *pl*TrpB. As a reference, standard samples were used, which contained 100 mM potassium phosphate (pH 7.5), 180 mM KCl, and 500 μM indole or 2 mM l‐tryptophan, respectively. Further HPLC‐based enzyme assays were performed to unravel alternative functions of *pl*TrpB and *pl*TrpA: To identify possible additional nucleophiles that might be preferred over indole, the same assay was performed in the absence of indole and in the presence of 500 μM of the tested compound. Either 10 μM *pl*TrpB, 10 μM *pl*TrpA, or 10 μM *pl*TrpA:*pl*TrpB were used to start the reaction. In a third approach, the reversed reaction starting with l‐tryptophan was assessed, again using the same assay including 10 μM *pl*TrpB but no l‐serine and no indole. Following incubation at 30°C and 500 rpm for 60 min, all reactions were stopped by centrifugation using a filter tube with a pore size of 10 kDa to remove any enzymes. The reaction products were subsequently analyzed by reversed‐phase HPLC using an Agilent system (1100 series) with an Eclipse XDB‐C18 (4.6 × 150) column. The separation was performed at 20°C with a flow rate of 0.25 mL/min using 0.1% formic acid in water as buffer A and 0.1% formic acid in acetonitrile as buffer B (gradient: 5%–100% buffer B).

### 4.5 Steady‐state kinetic measurements of TrpB enzymes

To determine TrpB activity, the difference in absorbance between indole and l‐tryptophan was used (Δ*ε*_290_ = 1890 M^−1^ cm^−1^) (Faeder & Hammes, 1970). Reactions were performed in triplicates at 30°C, and changes in absorbance were monitored using a spectrophotometer (JASCO V‐750). The experimental conditions included 50 mM potassium phosphate (pH 7.5), 180 mM KCl, 40 μM PLP, saturating concentrations of one substrate (indole/l‐serine) and varying concentrations of the other substrate (l‐serine/indole). When a constant baseline absorbance was reached, reactions were initiated by the addition of 0.5–7 μM of *pl*TrpB or *pl*TrpB‐con. For the TrpB reactions in the presence of *pl*TrpA, complex formation was induced by the addition of a 1.5‐fold molar excess of *pl*TrpA to 0.5–3 μM of *pl*TrpB or *pl*TrpB‐con prior to the start of the reaction. Initial velocities (*v*_i_) were calculated from the initial linear part of the resulting curve via division by Δ*ε*_290_. The determined reaction velocities were then normalized to the applied enzyme concentration (*v*_i_/*E*_0_) and plotted against the substrate concentration. The Michaelis constant *K*_M_ and the turnover number *k*_cat_ were obtained by fitting the data to the Michaelis–Menten equation using Origin 2022 (© OriginLab Corporation).

### 4.6 Determination of apparent *K*_d_^app^ values by activity titrations

To determine apparent *K*_d_^app^ values for the *pl*TrpA:*pl*TrpB and *pl*TrpA:*pl*TrpB‐con complexes, activity titrations were performed where TrpB activity was monitored as described above. Reactions were performed in duplicates at 30°C. The experimental conditions included 2 μM *pl*TrpB or *pl*TrpB‐con, 50 mM potassium phosphate (pH 7.5), 180 mM KCl, 40 μM PLP, saturating concentrations of both substrates (75 mM l‐serine, 1 mM indole) and varying concentrations of *pl*TrpA. The initial velocities measured for *pl*TrpB or *pl*TrpB‐con in the absence of *pl*TrpA were used for normalization and subtracted from all data points recorded in the presence of *pl*TrpA. The initial velocities were plotted as a function of the added *pl*TrpA concentration. To determine the apparent *K*_d_,^app^ the resulting data points were fitted to the data with a hyperbolic saturation curve using Origin 2022 (© OriginLab Corporation).

### 4.7 Determination of complex formation by analytical size‐exclusion chromatography

Analytical size exclusion chromatography was performed with *pl*TrpA (75 μM), *pl*TrpB (50 μM), and *pl*TrpB‐con (50 μM) that were applied individually or as a mixture between *pl*TrpA and *pl*TrpB or *pl*TrpB‐con, respectively, to a Superdex200 increase 10/300 GL column (GE Healthcare) operated on an ÄKTAmicro system (GE Healthcare). The column was equilibrated with 50 mM potassium phosphate (pH 7.5), 300 mM KCl, and 75 mM l‐serine at 25°C. Protein elution was performed at a flow rate of 0.3 mL/min and was followed by absorbance measurements at 280 nm. Calibration was performed with the Cytiva LMW and HMW calibration kits.

### 4.8 Circular dichroism (CD) spectroscopy

To assess the structural integrity of the proteins, far‐UV CD spectroscopy was used. Spectra were recorded with a CD spectrometer (J‐815, JASCO) between 280 and 190 nm using a quartz cuvette (0.2 mm). Measurements were conducted at 25°C in five replicas. All spectra were corrected for buffer absorption (50 mM KP, pH 7.5; 300 mM KCl) and smoothed using the Savitzky–Golay algorithm (Savitzky & Golay, 1964) (convolution width 7) implemented in the Spectra Analysis software provided by JASCO. The mean molar ellipticity per residue *θ*_MRW_ (deg cm^2^ dmol^−1^) was calculated from the observed ellipticity *θ*_obs_ (mdeg), the pathlength of the cuvette *d* (cm), the protein concentration *c* (μM), and the number of residues *N*_A_, according to the following equation:

$$\theta_{\mathrm{MRW}}=\frac{\theta_{\mathrm{obs}}\times 10^{5}}{c\times d\times N_{A}}.$$

### 4.9 Analysis of the genomic neighborhood of *pl*TrpA and *pl*TrpB

Genome neighborhood diagrams were retrieved from the Enzyme‐Function‐Initiative Genome‐Neighborhood‐Tool (EFI‐GNT) (Oberg et al., 2023; Zallot et al., 2019) using the amino acid sequence of either *pl*TrpA or *pl*TrpB as input. The genes flanking *pltrpA* and *pltrpB* were analyzed regarding their functional annotation.

### 4.10 Multiple sequence alignment (MSA) and identification of *pl*TrpB

All 6373 sequences that are annotated as TrpB1 enzymes in the KEGG (Kanehisa & Goto, 2000) database were retrieved and used to generate a multiple sequence alignment with MAFFT (Katoh & Standley, 2013). To identify proteins which contain the LBCA‐Res_6_, the MSA was iteratively filtered by discarding any proteins which did not contain one of the six residues. This shrunk the dataset to only one protein variant that contained all six LBCA‐Res_6_ residues. For mutational planning of the Res_6_ residues in *pl*TrpB, we analyzed the MSA in JalView (Waterhouse et al., 2009) and exchanged the LBCA‐Res_6_ to the consensus at each position (E46A, E52D, T63S, S197T, A289G, M290N) giving rise to *pl*TrpB‐con. In the case of E46, the Res_6_ residue of *pl*TrpB wild‐type represented the most commonly found amino acid. The corresponding position was occupied by an Ala in Anc3TrpB, which is highly dependent on TrpA. Therefore, and because Ala is the second most commonly found amino acid at this position, we replaced Glu with Ala.

### 4.11 Molecular modeling system preparation

The starting structures for the two systems (the *pl*TrpA:*pl*TrpB and *pl*TrpA:*pl*TrpB‐con) were generated with the multimer version of the AlphaFold2 (AF2) (Jumper et al., 2021) neural network. The AF2 predicted structures are available at https://doi.org/10.5281/zenodo.14872182. The AF2 models simulated had a predicted LDDT‐Cα score (pLDDT) higher than 92. To generate the TrpB homodimer enzyme, the TrpA subunits of the predicted structures were removed. The aminoacrylate (AA) Schiff base and quinonoid (QQ2) intermediates were placed in the TrpB subunits through superposition to the external aldimine 2 (Aex2) intermediate of the engineered TrpB crystal structure with PDB accession code 6AM8. Also, to avoid clashes with the QQ2 intermediate, the chi2, chi3, and chi4 torsion angles of the catalytic lysine (i.e., Lys84) were switched to the ones in the 6AM8 x‐ray structure.

The water molecules added to each homodimer were selected from the DBSCAN clusterization (Ester et al., 1996; Jukič et al., 2017) algorithm implemented in the scikit‐learn Python library (Pedregosa et al., 2011), of different x‐ray TrpB monomers. Additionally, three conserved sodium ions in the crystal structures were added to all structures located at the dimer interface and in each monomer close to the active site.

The MD parameters for QQ2 and AA intermediates were generated with the antechamber and parmchk2 modules of AMBER22 (Case et al., 2022) using the second generation of the general amber force field (GAFF2) (Case et al., 2022; Wang et al., 2004). The intermediates were optimized at the B3LYP/6‐31G(d) level of theory, including Grimme's dispersion correction with Becke–Johnson Damping (D3‐BJ) and the polarizable conductor model (PCM) (dichloromethane, *ε* = 8.9) as an estimation of the dielectric permittivity in the enzyme active site (Schutz & Warshel, 2001). The partial charges (RESP model) (Bayly et al., 1993) were set to fit the electrostatic potential generated at the HF/6‐31G(d) level of theory. The charges were calculated according to the Merz–Singh–Kollman (Besler et al., 1990; Singh & Kollman, 1984) scheme using the Gaussian16 software package (Frisch et al., 2016). The protonation states were predicted using PROPKA (Olsson et al., 2011; Søndergaard et al., 2011). However, the protonation state of the catalytic residue Lys84 was neutral (i.e., LYN84), as is described in the mechanism at the QQ2 intermediate. The enzyme structures were solvated in a pre‐equilibrated system using the OPC water model and neutralized by the addition of explicit counterions (i.e., Na^+^) using the AMBER22 leap module. All MD simulations were performed using the ff19SB force field.

### 4.12 MD simulation details

MD equilibration phase was done following the protocol described by Roe and Brooks with small differences fine‐tuned to our systems (Roe & Brooks, 2020). The bonds involving hydrogen are constrained by the SHAKE algorithm during the non‐minimization steps. Long‐range electrostatic effects were modeled using the particle mesh‐Ewald method (Darden et al., 1993). For Lennard‐Jones and electrostatic interactions, a 10 Å cut‐off was applied. The MD protocol starts with the minimization phase of 1500 steps of the steepest descent method followed by 3500 steps of the conjugate gradient method with a positional restraint (i.e., a force constant of 5.0 kcal mol^−1^ Å^−2^) to the protein heavy atoms. In the following heating phase, a temperature increment from 25 to 300 K during 20 ps of MD simulation time, a Langevin thermostat with a collision frequency of 5 ps^−1^, and a positional restraint (i.e., a force constant of 5.0 kcal mol^−1^ Å^−2^) to the protein heavy atomsis performed. A minimization and heating of all atoms in the system is the following step. This starts with two minimization stages of 1000 steps of the steepest descent method followed by 1500 steps of the conjugate gradient method, each with a positional restraint (i.e., force constant of 2.0 kcal mol^−1^ Å^−2^ in the first minimization and 0.1 kcal mol^−1^ Å^−2^ in the second) to the protein heavy atoms. Following this, a third minimization phase of 1500 steps of the steepest descent method followed by 3500 steps of the conjugate gradient method without any positional restraint is performed. The system is then heated in accordance with the previously established procedure. Finally, a five‐round equilibration phase at the NPT ensemble with a constant pressure of 1 atm is performed. The first four rounds were done with the Berendsen barostat, whereas the fifth one was done with a Monte Carlo barostat. For all equilibration rounds, a Langevin thermostat with a collision frequency of 1 ps^−1^ was used. A positional restraint to the protein heavy atoms with a force constant of 1.0 and 0.5 kcal mol^−1^ Å^−2^ was applied to the first and second equilibration rounds, respectively. In the third round of 10 ps equilibration, a positional restraint to the backbone heavy atoms with a force constant of 0.5 kcal mol^−1^ Å^−2^ was used. The fourth and fifth equilibration rounds of 10 ps and 1 ns, respectively, were performed without any restraint. The production runs were performed at the NVT ensemble with the Langevin thermostat with a collision frequency of 1 ps^−1^ during 50 ns for all systems. A total of 20 replicas of equilibration and production runs were performed, reaching a total simulation time of 1 μs/system (20 replicas × 50 ns) for systems *pl*TrpB, *pl*TrpA:*pl*TrpB, *pl*TrpB‐con, and *pl*TrpA:*pl*TrpB‐con, with QQ2 or AA intermediates, leading to a final number of 8 systems. The MD trajectories were analyzed using the Python packages MDTraj (McGibbon et al., 2015), pytraj (Nguyen et al., 2016) which is part of the cpptraj package (Roe & Brooks, 2020), MDAnalysis (Gowers et al., 2016), and PyEMMA (Scherer et al., 2015).

## AUTHOR CONTRIBUTIONS

**Thomas Kinateder:** Conceptualization; writing – original draft; methodology; investigation. **Lukas Drexler:** Conceptualization; investigation; writing – original draft; methodology. **Cristina Duran:** Investigation; methodology; writing – review and editing; software. **Sílvia Osuna:** Conceptualization; funding acquisition; supervision; writing – review and editing; software; project administration. **Reinhard Sterner:** Conceptualization; funding acquisition; writing – review and editing; supervision; project administration.

## Supporting information


**Data S1** Supporting Information.

## Data Availability

The data that support the findings of this study are available from the corresponding author upon reasonable request.
