# Supplementary material for: A naturally occurring standalone TrpB enzyme provides insights into allosteric communication within tryptophan synthase
Source: Protein Sci. 2025 Mar 18;34(4):e70103. doi: 10.1002/pro.70103 (PMC11917138; doi:10.1002/pro.70103)
Supplement: Supplementary file 1 — Data S1 Supporting Information. [file PRO-34-e70103-s001.pdf]

Supporting Information

A naturally occurring stand-alone TrpB enzyme provides insights into allosteric communication within tryptophan synthase

Thomas Kinateder[1#], Lukas Drexler[1#], Cristina Duran[2], Sílvia Osuna[2,3], Reinhard Sterner[1]**

[1]Institute of Biophysics and Physical Biochemistry, Regensburg Center for Biochemistry, University of Regensburg, D-93040 Regensburg, Germany.

[2]CompBioLab Group, Institut de Química Computacional i Catàlisi (IQCC) and Departament de Química, Universitat de Girona, Girona 17003, Spain.

[3]ICREA, Barcelona 08010, Spain.

***Corresponding Authors:**
Sílvia Osuna: Email: silvia.osuna@udg.edu
Reinhard Sterner: Email: reinhard.sterner@ur.de

[#] Thomas Kinateder and Lukas Drexler contributed equally to this work.

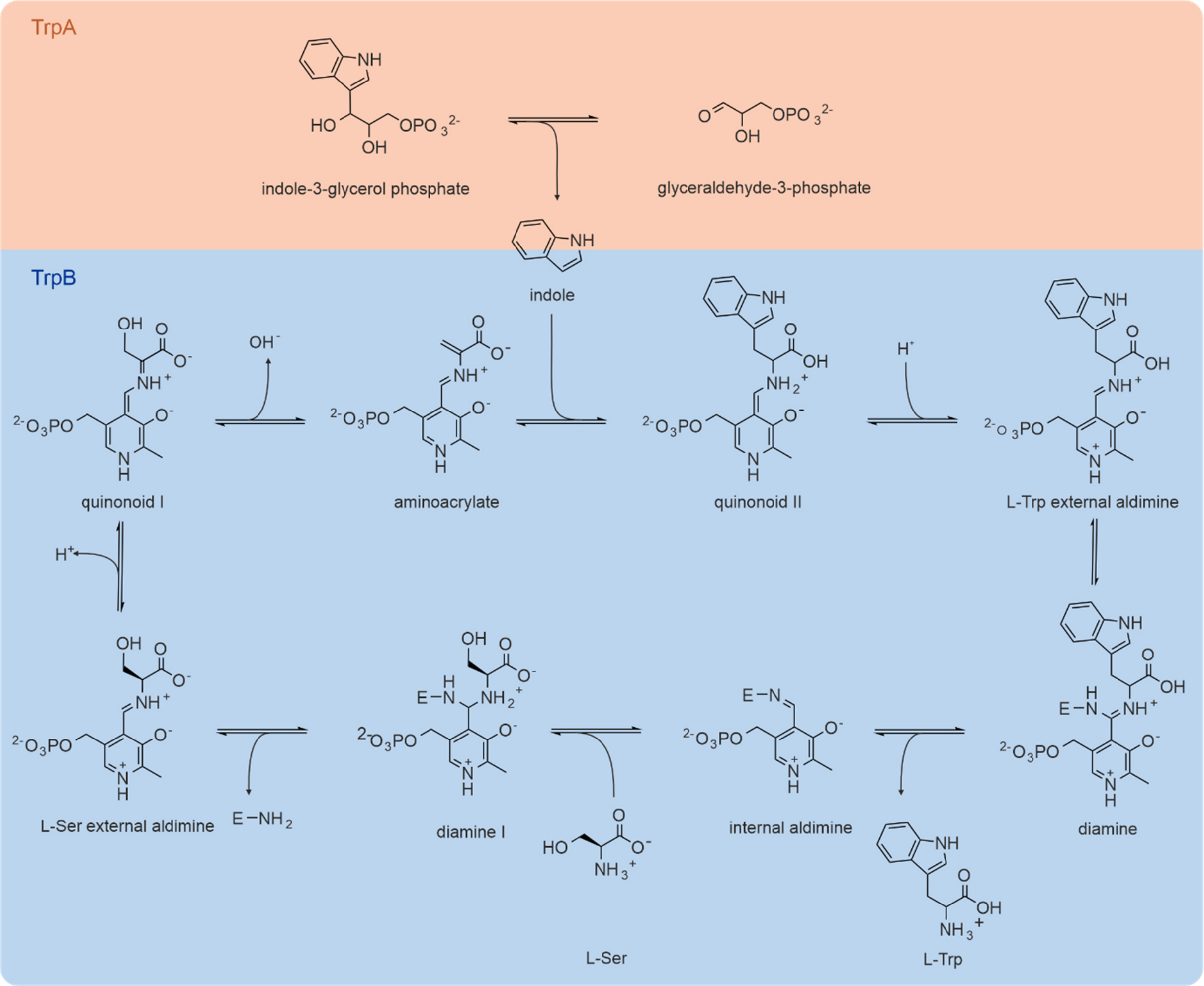

Figure S1. Partial reactions catalyzed by TrpA and TrpB [1]. TrpA (orange panel) catalyzes the retro-aldol cleavage of indole-3-glycerol phosphate to glyceraldehyde-3-phosphate and indole. The latter is transferred to TrpB. The reaction cycle of TrpB (blue panel) starts at the internal aldimine, which consists of a pyridoxalphosphate (PLP) cofactor that is covalently bound to a lysine residue. Then, L-serine (L-Ser) attacks the internal aldimine and diamine I is formed. Subsequently, the lysine residue is released, giving rise to the external aldimine with bound L-Ser. Afterwards, a two-step elimination of water first leads to quinonoid I which is followed by the formation of an aminoacrylate. The aminoacrylate then reacts with the incoming indole from TrpA to form quinonoid II and, upon loss of a hydrogen atom, an external aldimine with L-tryptophan (L-Trp) is formed. This external aldimine reacts with the enzyme-bound lysine residue to form another diamine. Finally, L-Trp is released, restoring the internal aldimine.

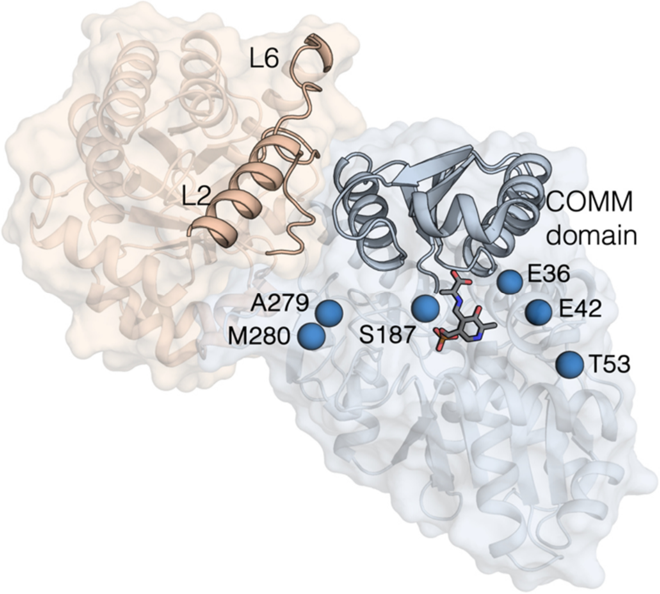

Figure S2. Location of the Res_6 mutations in the *pl*TrpA:*pl*TrpB complex. E36 and E42 are located at the surface, T53 at the β-β interface, S187 at the active site, and A279 as well as M280 at the α-β interface. *Pl*TrpA is colored in orange, whereas *pl*TrpB is colored in blue. The allosterically and catalytically relevant loops/domains are highlighted and labeled: The two active site loops L6 and L2 in *pl*TrpA, and the COMM domain in *pl*TrpB.

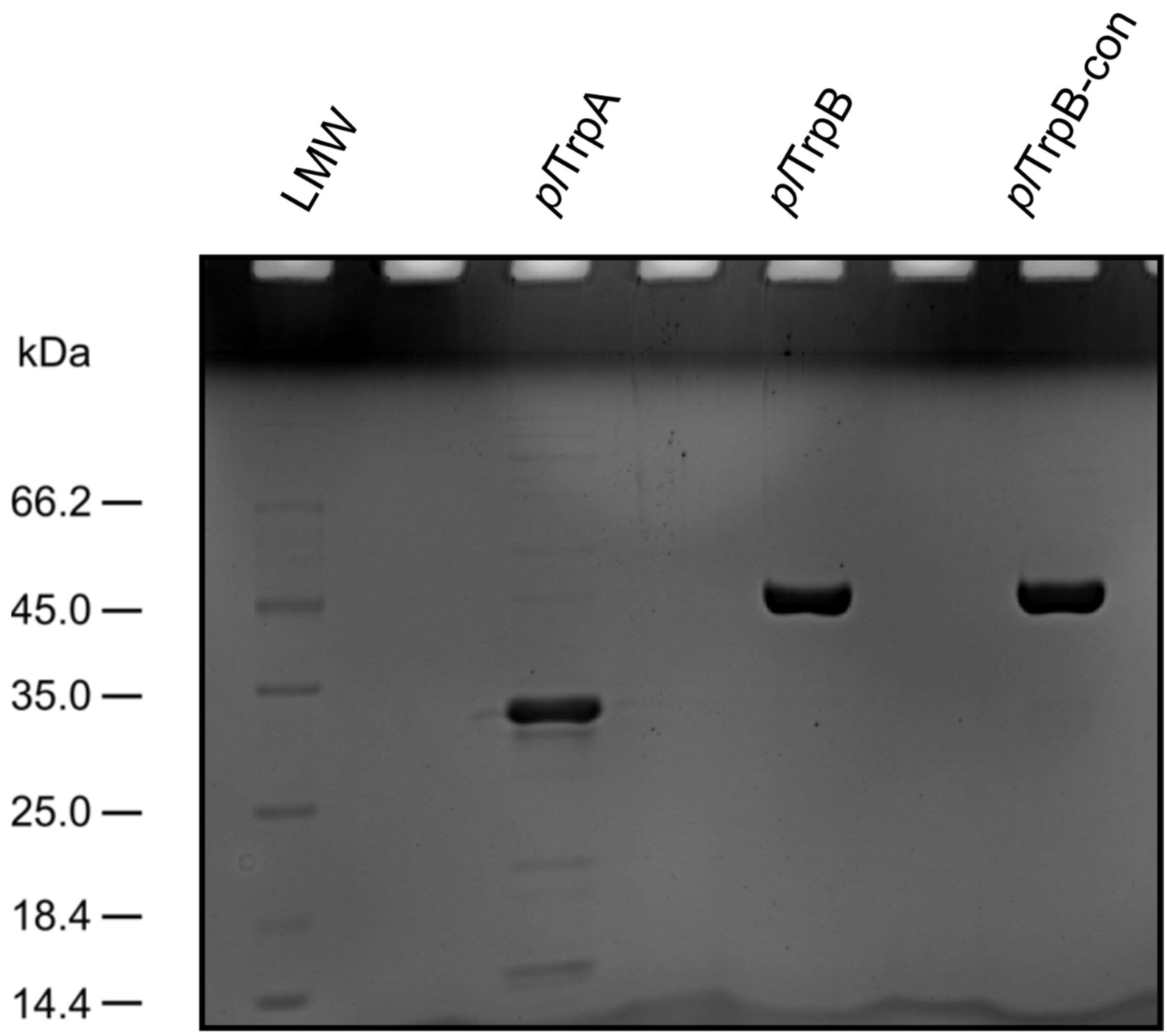

Figure S3. Assessment of the purity of *pf*TrpA, *pf*TrpB, and *pf*TrpB-con. After enrichment by IMAC and SEC, the purity of the proteins (3µg each) was assessed by SDS-PAGE. LMW protein standard (Thermo Fisher Scientific) was used to estimate the molecular weight of the purified proteins, which are consistent with the theoretical values for *pf*TrpA (30.5 kDa), *pf*TrpB (43.9 kDa), and *pf*TrpB-con (43.8 kDa) as calculated using the Protparam tool [2].

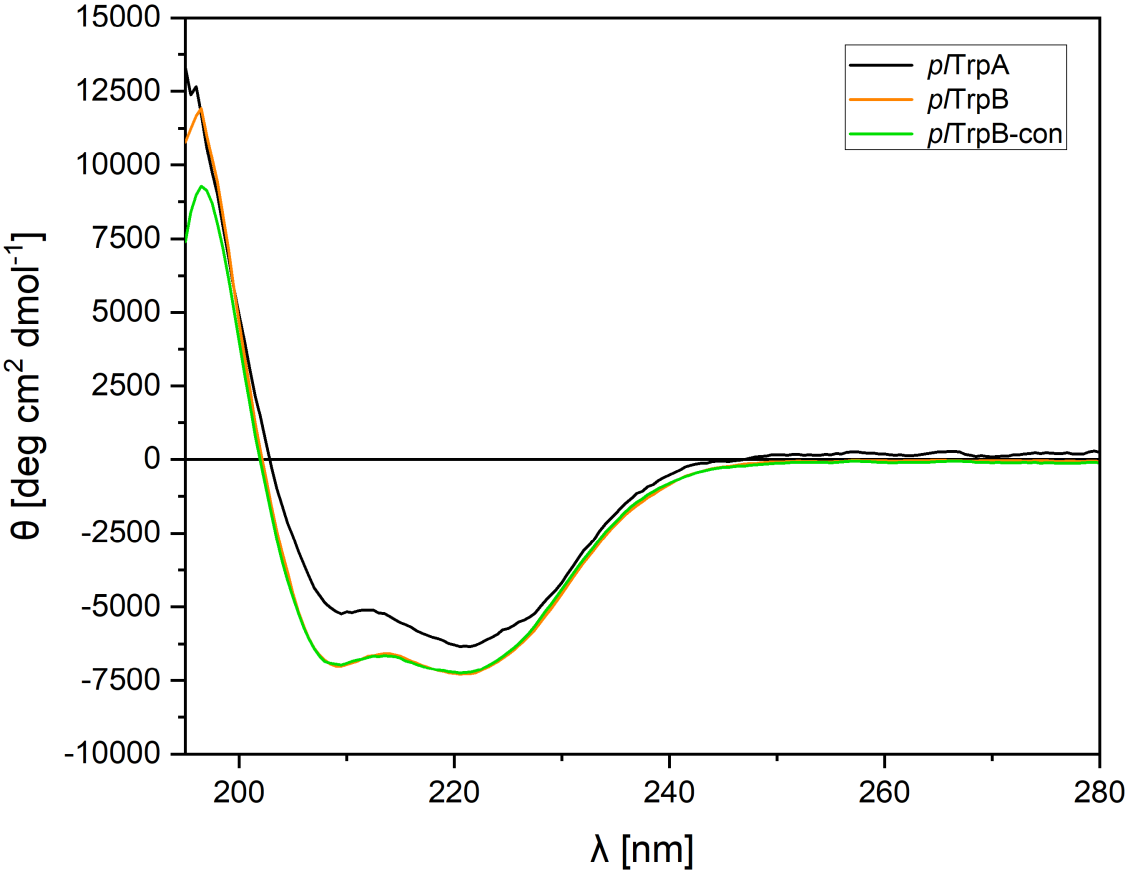

Figure S4. Assessment of secondary structure of purified *pl*TrpA, *pl*TrpB, and *pl*TrpB-con. Far-UV CD spectra of 20 µM protein each were recorded in 50 mM KP (pH 7.5), 300 mM KCl. Measurements were conducted in five replicas at 25 °C using a quartz cuvette (d = 0.2 mm). *pl*TrpA, *pl*TrpB, and *pl*TrpB-con show CD spectra that are characteristic of properly folded proteins. No significant differences between the spectra of *pl*TrpB and *pl*TrpB-con were observable.

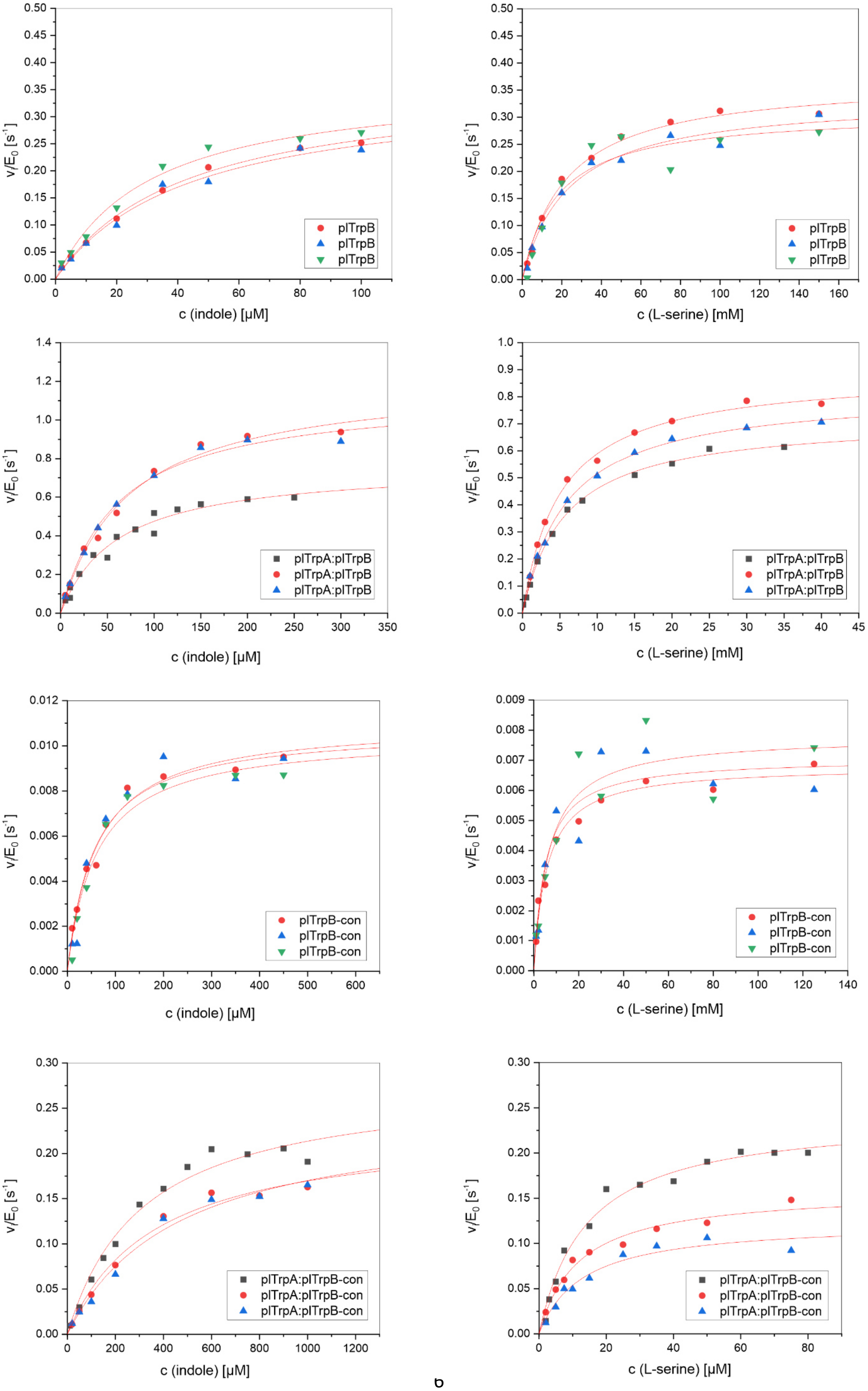

v/E0 [s-1]
c (indole) [µM]
c (L-serine) [mM]
c (L-serine) [µM]
plTrpB
plTrpB
plTrpB
plTrpA:plTrpB
plTrpA:plTrpB
plTrpA:plTrpB
plTrpB-con
plTrpB-con
plTrpB-con
plTrpA:plTrpB-con
plTrpA:plTrpB-con
plTrpA:plTrpB-con

		measurement 1		measurement 2		measurement 3	
**Protein**	**substrate**	**k_{cat} [s^{-1}]**	**K_M [µM]**	**k_{cat} [s^{-1}]**	**K_M [µM]**	**k_{cat} [s^{-1}]**	**K_M [µM]**
*pl*TrpB	L-serine	0.3366	23325	0.3706	22184	0.3096	17685
	indole	0.3659	31	0.3657	42	0.3546	44
*pl*TrpA:*pl*TrpB	L-serine	0.7201	5687	0.8915	5130	0.8202	5929
	indole	0.7639	61	1.2249	73	1.1300	61
*pl*TrpB-con	L-serine	0.0071	5156	0.0068	5781	0.0078	6496
	indole	0.0111	60	0.0108	56	0.0105	61
*pl*TrpA:*pl*TrpB-con	L-serine	0.2418	14	0.1589	12	0.1237	13
	indole	0.2805	314	0.2300	362	0.2486	462

Average ± standard deviation	k_{cat} [s^{-1}]	K_M^{Ser} [mM]	K_M^{Ind} [µM]	k_{cat}/K_M^{Ser} [s^{-1} M^{-1}]	k_{cat}/K_M^{Ind} [s^{-1} M^{-1}]
***pl*TrpB**	0.35 ± 0.02	21 ± 3	39 ± 7	17 ± 2.6	8970 ± 1720
***pl*TrpA:*pl*TrpB**	0.93 ± 0.21	5.6 ± 0.4	65 ± 7	166 ± 39	14300 ± 3510
***pl*TrpB-con**	0.009 ± 0.002	5.8 ± 0.7	59 ± 3	1.6 ± 0.39	153 ± 34
***pl*TrpA:*pl*TrpB-con**	0.21 ± 0.06	0.013 ± 0.001	380 ± 76	16200 ± 4770	553 ± 193

Figure S5. Steady-state enzyme kinetics of *pl*TrpB and *pl*TrpB-con in isolation and in complex with *pl*TrpA. Reactions were performed in triplicates at 30°C. The experimental conditions included 50 mM potassium phosphate (pH 7.5), 180 mM KCl, 40 µM PLP, saturating concentrations of one substrate (indole/L-serine) and varying concentrations of the other substrate (L-serine/indole). The Michaelis constant K_M and the turnover number k_{cat} were obtained by fitting the data to the Michaelis-Menten equation using Origin 2022 (© OriginLab Corporation). Average and standard deviation for k_{cat} (average of 6 values), K_M (average of 3 values), and k_{cat}/K_M were calculated from the three independent measurements.

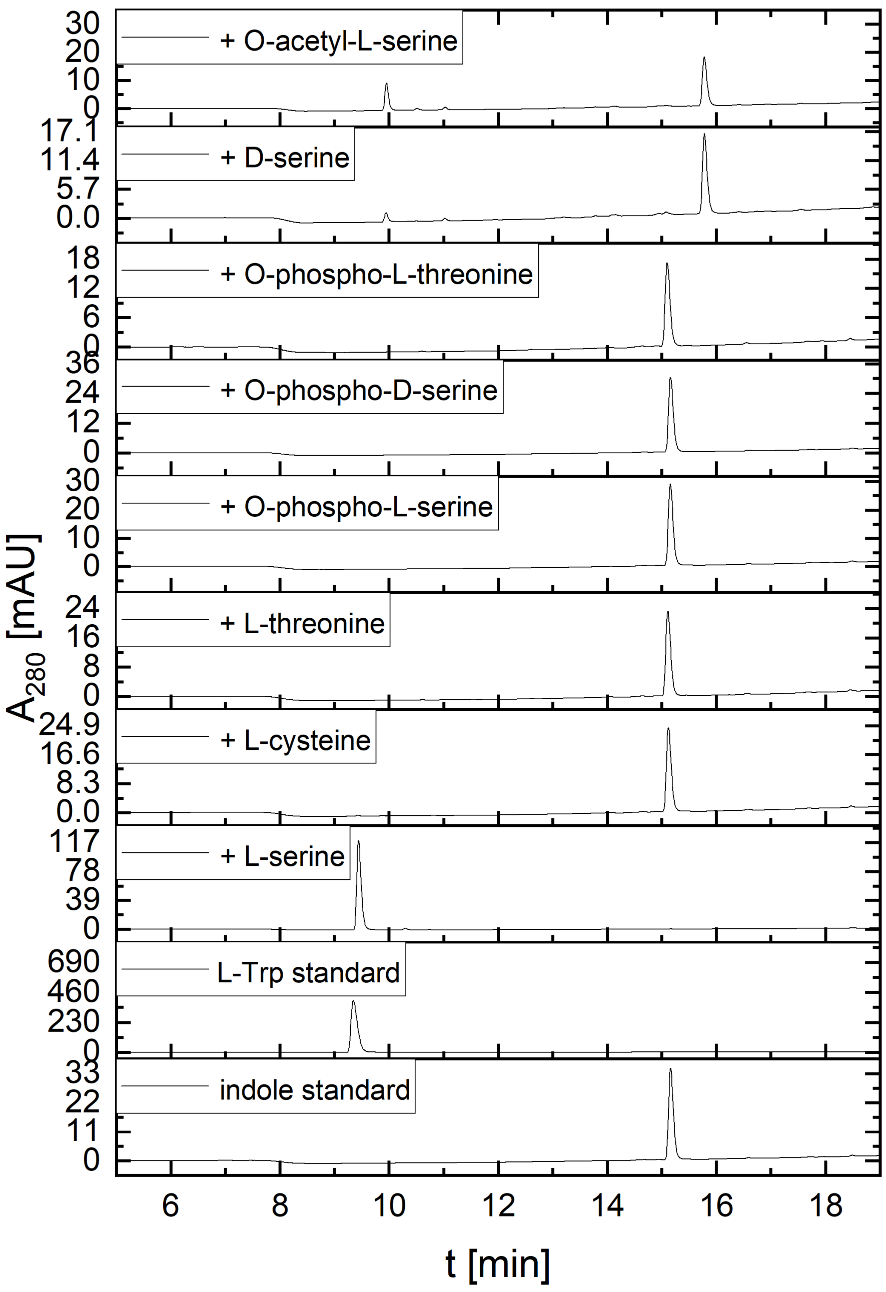

Figure S6. HPLC-based screening with potential alpha-amino acid substrates of *pl*TrpB. All enzymatic assays contained 500 µM indole, 100 mM potassium phosphate (pH 7.5), 180 mM KCl, 40 µM PLP, 2 mM of the respective alpha-amino acid, and 5 µM *pl*TrpB. Standard samples contained 100 mM potassium phosphate (pH 7.5), 180 mM KCl, and 500 µM indole or 2 mM L-tryptophan, respectively. All samples were incubated for 60 min at 30 °C while shaking at 500 rpm on an Eppendorf table top shaker. Only in the presence of L-serine complete turnover of indole to L-tryptophan was detected, while in the presence of D-serine and O-acetyl-L-serine partial turnover was observed. However, the observed formation of L-Trp in the presence of D-serine could also be due to racemization of D-serine in water with accompanying formation of L-serine. These results suggest L-serine being the main alpha-amino acid substrate of *pl*TrpB.

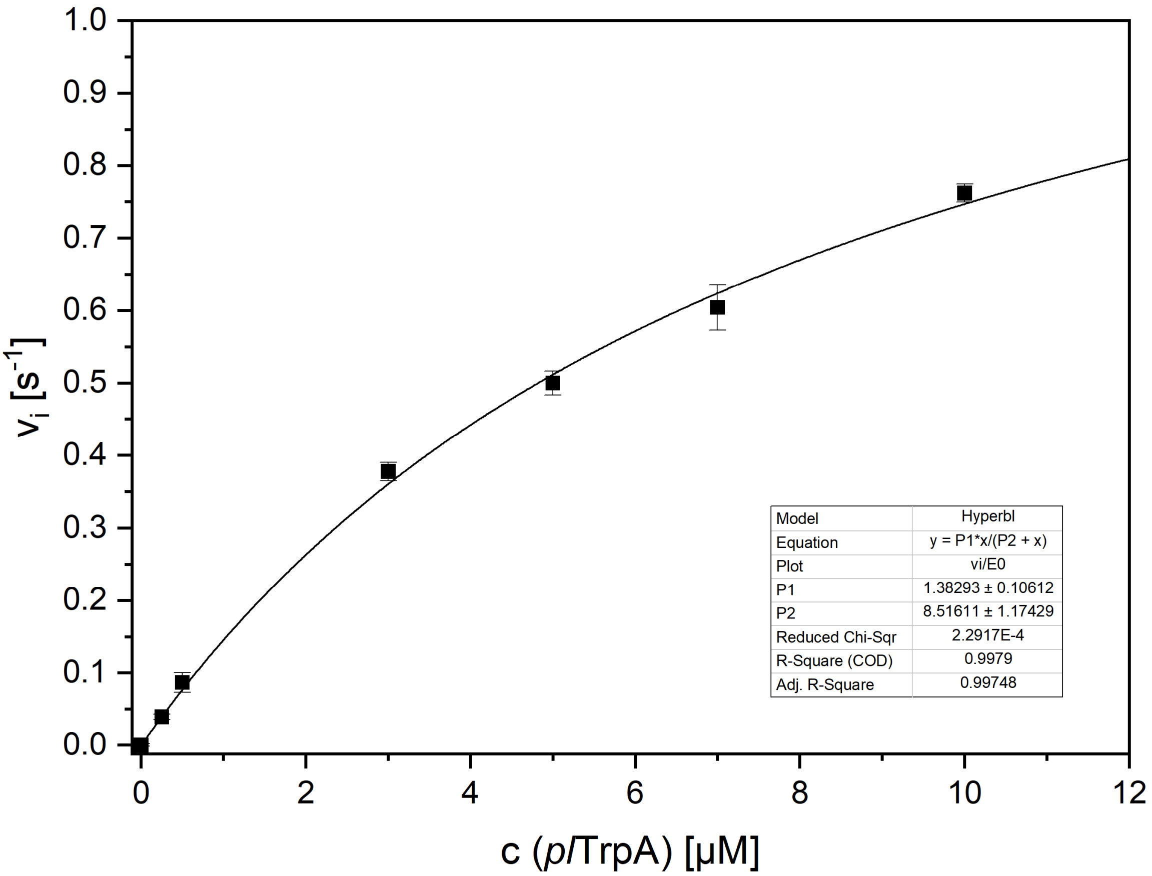

Figure S7. Activity titration for the determination of an apparent dissociation constant (K_d^{app}) for formation of the *pf*TrpA:*pf*TrpB complex. Reactions were performed in duplicates at 30°C. The experimental conditions included 2 µM *pf*TrpB, 50 mM potassium phosphate (pH 7.5), 180 mM KCl, 40 µM PLP, saturating concentrations of both substrates (75 mM L-serine, 1 mM indole) and varying concentrations of *pf*TrpA. The activity measured for *pf*TrpB in absence of *pf*TrpA was subtracted from all data points recorded in presence of *pf*TrpA. An apparent K_d^{app} of 8.5 µM was determined by fitting the data to a hyperbolic function.

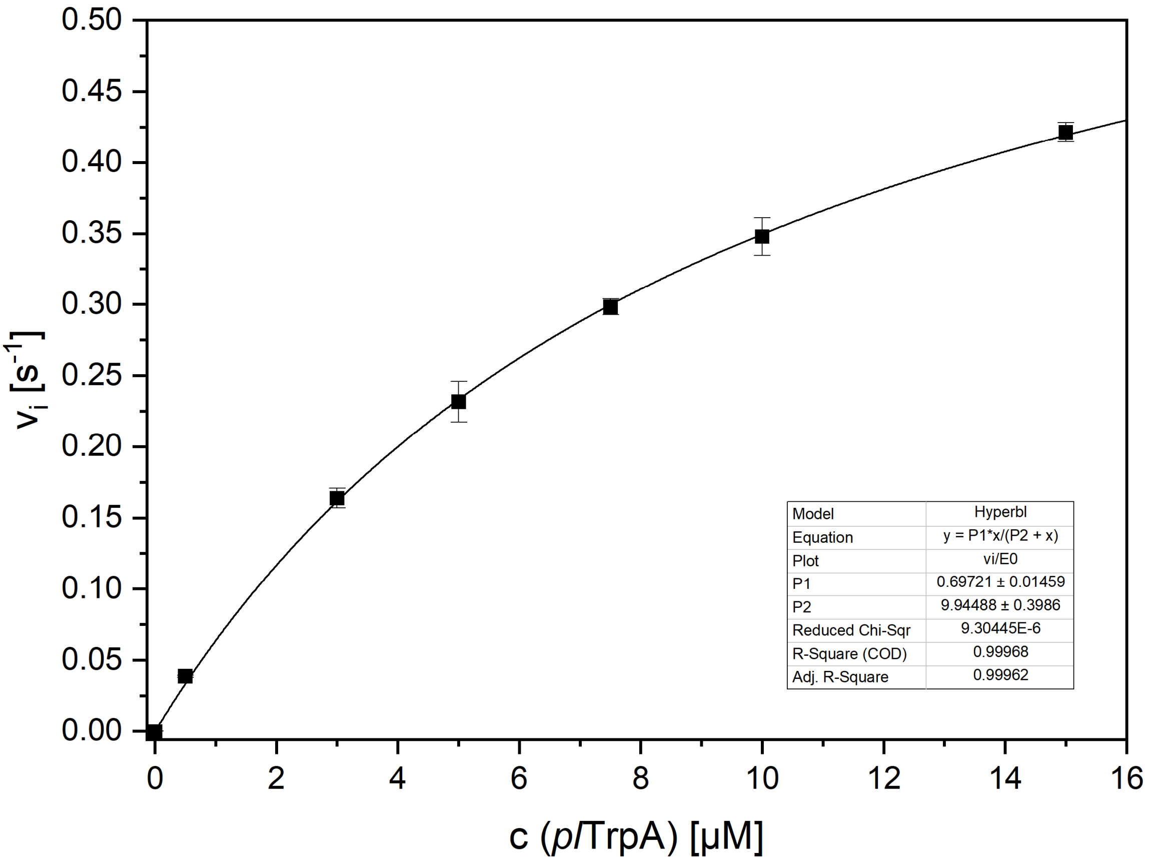

Figure S8. Activity titration for the determination of an apparent dissociation constant (K_d^{app}) for formation of the *pf*TrpA:*pf*TrpB-con complex. Reactions were performed in duplicates at 30°C. The experimental conditions included 2 µM *pf*TrpB-con, 50 mM potassium phosphate (pH 7.5), 180 mM KCl, 40 µM PLP, saturating concentrations of both substrates (75 mM L-serine, 1 mM indole) and varying concentrations of *pf*TrpA. The activity measured for *pf*TrpB-con in absence of *pf*TrpA was subtracted from all data points recorded in presence of *pf*TrpA. An apparent K_d^{app} of 9.9 µM was determined by fitting the data to a hyperbolic function.

A

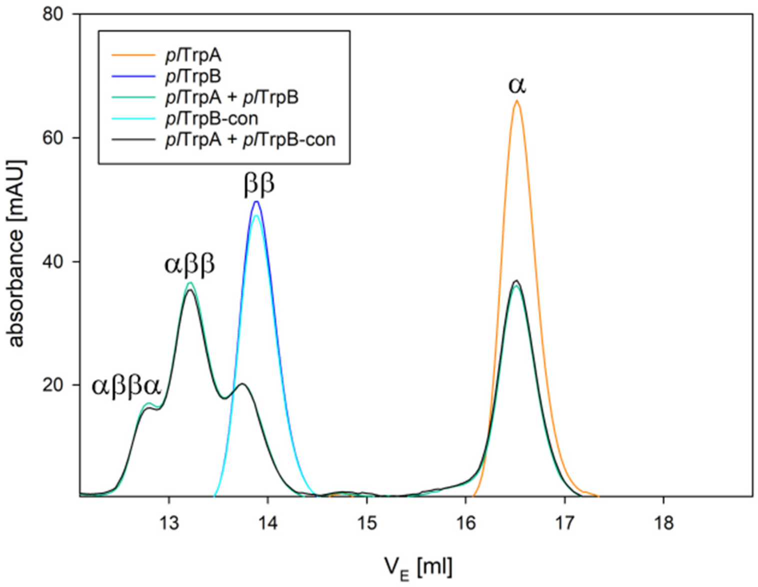

B

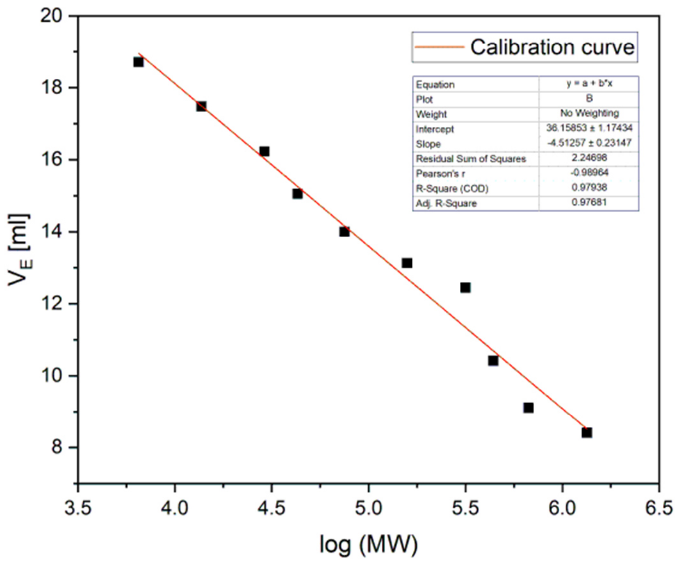

C

Enzymes	Oligomer	MW (theoretical) [Da]	Elution volume [ml]	MW (apparent) [Da]
*pf*TrpA	α	30461	16.52	22493
*pf*TrpB	ββ	87760	13.88	86510
*pf*TrpB-con	ββ	87326	13.88	86510
*pf*TrpA + *pf*TrpB	α	30461	16.51	22607
	ββ	87760	13.74	92915
	αββ	118221	13.21	121769
	αββα	148682	12.80	150105
*pf*TrpA + *pf*TrpB-con	α	30461	16.51	22607
	ββ	87326	13.74	92915
	αββ	117787	13.21	121769
	αββα	148248	12.79	150873

Figure S9. (**A**) Analytical size-exclusion chromatography (Superdex S200 column) was performed at 25°C with the indicated proteins using 50 mM potassium phosphate (pH 7.5), 300 mM KCl, and 75 mM L-serine as running buffer. The elution volumes V_E were determined by measuring protein absorbance at 280 nm. (**B**) The elution volumes of standard proteins with known molecular weight (MW) were used to generate a calibration curve. (**C**) Apparent molecular weights and oligomerization states were calculated using the observed elution volumes and the calibration curve. *pf*TrpA alone (applied at a concentration of 75µM) showed a peak at an elution volume that corresponds to the expected molecular weight of the isolated α-monomer whereas both *pf*TrpB (50 µM) and *pf*TrpB-con (50 µM) showed a peak at an elution volume that corresponds to the expected molecular weight of the ββ-dimer. A mixture of *pf*TrpA (75µM) with either *pf*TrpB (50µM) or *pf*TrpB-con (50µM) resulted in a total of four elution peaks in both cases, one with an elution volume corresponding to the expected molecular weight of the α-monomer, one corresponding to the ββ-dimer, one corresponding to the αββ-trimer, and one corresponding to the αββα-tetramer. The relative peak heights were nearly identical for both the mixture of *pf*TrpA with *pf*TrpB and the mixture of *pf*TrpA with *pf*TrpB-con, indicating that the mutations introduced into *pf*TrpB-con did not influence the dissociation constant for complex formation and hence the population of the different oligomeric states.

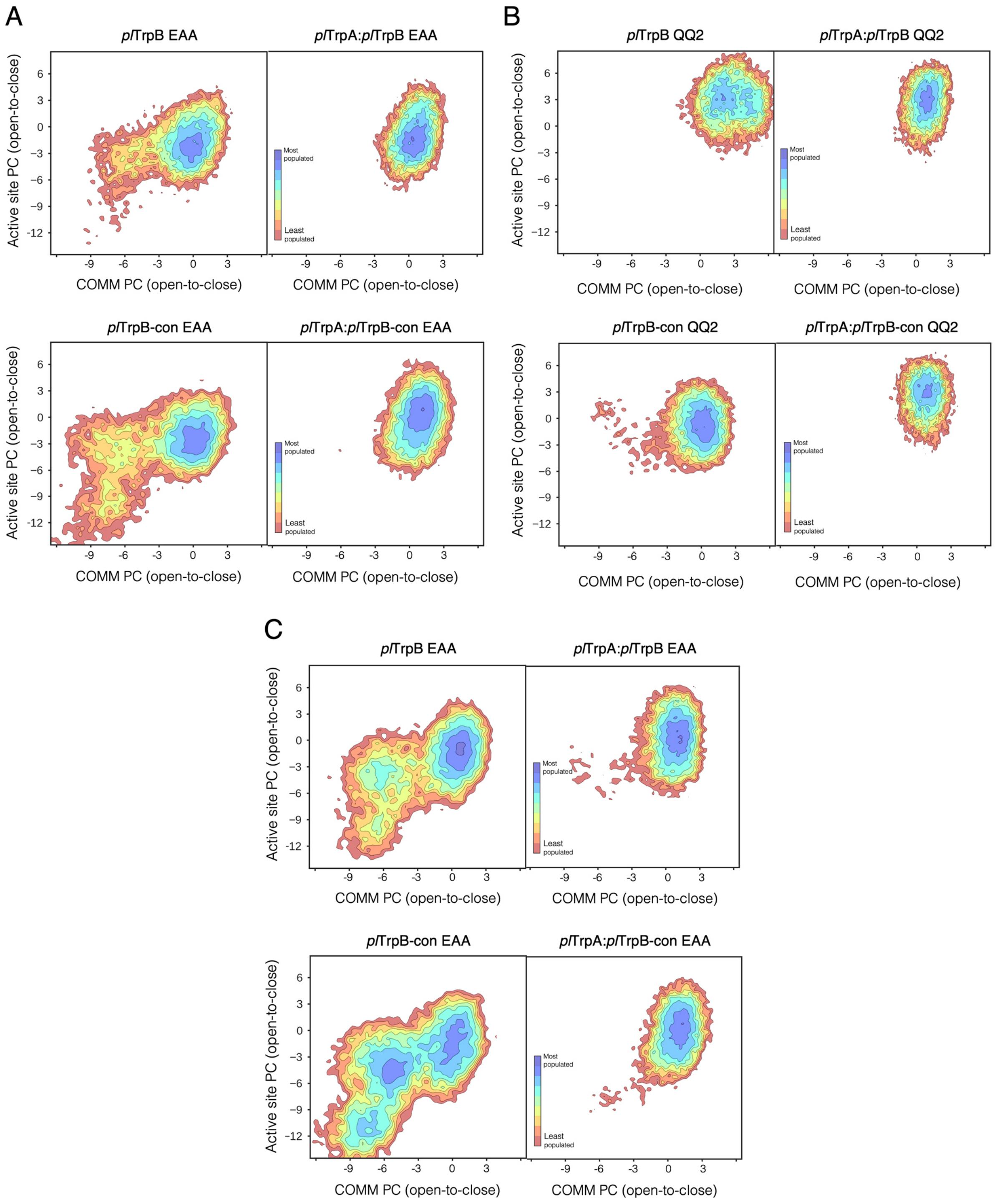

Figure S10. Reconstructed conformational landscapes of *pl*TrpB, *pl*TrpA:*pl*TrpB (top), *pl*TrpB-con and *pl*TrpA:*pl*TrpB-con (bottom) at the (**A**) aminoacrylate AA reaction intermediate with the protonated catalytic K82, (**B**) quinonoid QQ2 and (**C**) aminoacrylate AA reaction intermediates with the neutral catalytic K82. The PCA conformational landscape was generated considering distances between carbon alpha atoms of the residues included in the COMM domain (x-axis) and active site (y-axis). Negative values of PC correspond to open states and positive ones to closed states.

At the AA intermediate, despite the catalytic K82 presenting two different protonation states (A and C panels) the reconstructed conformational landscapes are similar, thus leading to the same conclusions. An overlay of the conformational landscapes of isolated *pf*TrpB (in color) and in complex with *pf*TrpA (in gray scale) is shown in the main text **Figures 3** and **4**.

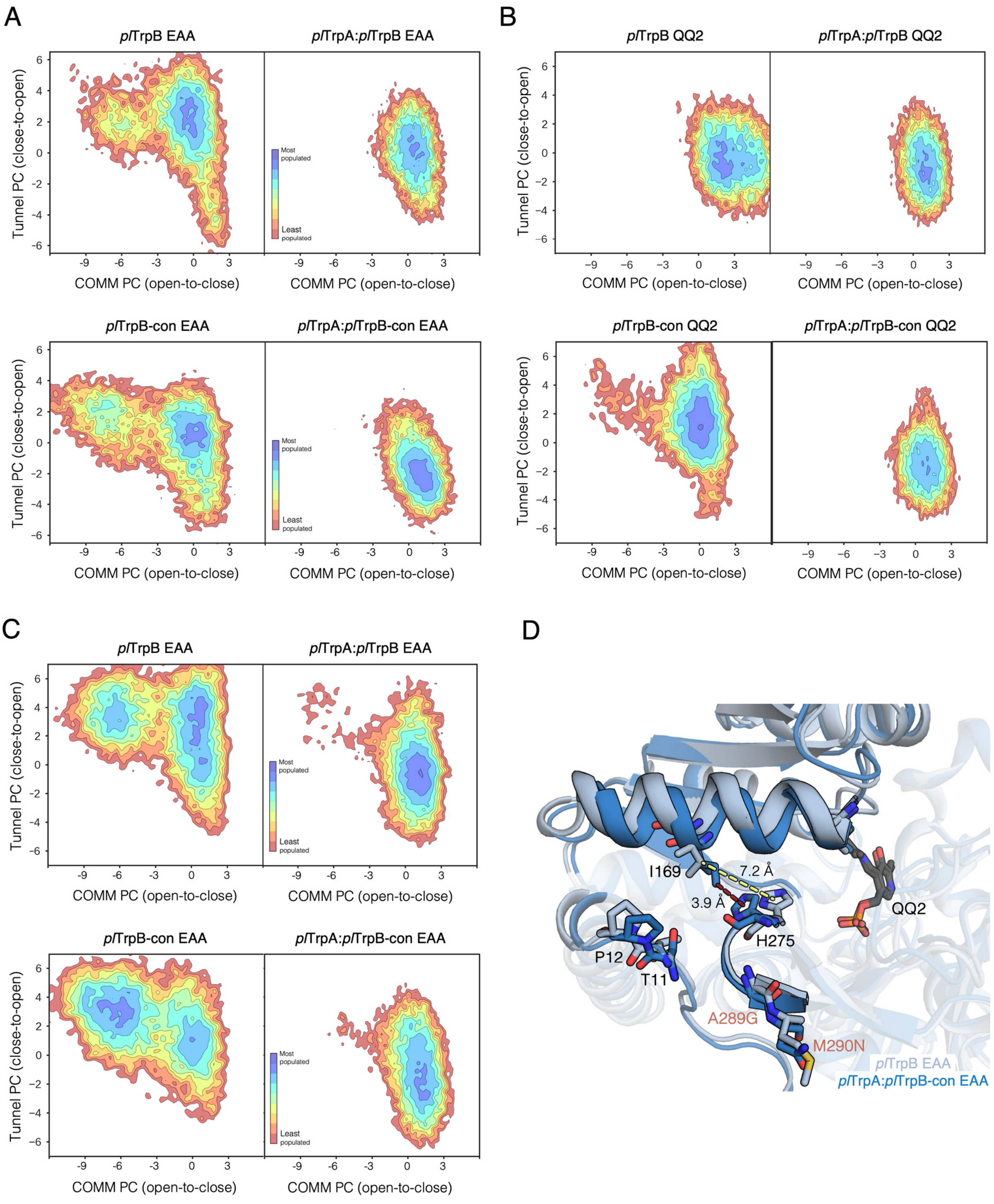

Figure S11. Reconstructed conformational landscapes of *pl*TrpB, *pl*TrpA:*pl*TrpB (top), *pl*TrpB-con and *pl*TrpA:*pl*TrpB-con (bottom) at the (**A**) aminoacrylate AA reaction intermediate with the protonated catalytic K82, (**B**) quinonoid QQ2 and (**C**) aminoacrylate AA reaction intermediates with the neutral catalytic K82. The PCA conformational landscape was generated considering distances between carbon alpha atoms of the residues included in the COMM domain (x-axis) and the tunnel (y-axis). Negative values of COMM PC correspond to open states, whereas positive to closed states. Negative values of tunnel PC correspond to

closed states, whereas positive to open states. (**D**) Overlay of most populated structures extracted from conformational landscapes in panel (A), representing the open (*i.e. pl*TrpB EAA) and closed (*i.e. pl*TrpA:*pl*TrpB-con EAA) states of the tunnel residues. The distance between H275 and I169 is represented in dashed lines as the most contributing distance in the tunnel PC (y axis). P12 and T11, which are key residues for indole channeling, are displayed in sticks. Two of the mutations introduced in *pl*TrpA-con variant, A289G and M290N (shown as sticks), are nearby the residues contributing to the tunnel gate.

At the AA intermediate, despite the catalytic K82 presenting two different protonation states (A and C panels) the reconstructed conformational landscapes are similar, thus leading to the same conclusions. The overlay of a representative conformation presenting the TrpA-TrpB tunnel either closed (taken from *pl*TrpA:*pl*TrpB-con) or open (isolated *pl*TrpB) shows that the closing of the tunnel is induced by a slight displacement of the helix containing I169, which gets positioned close to the H275, previously identified as important for tunnel formation [3].

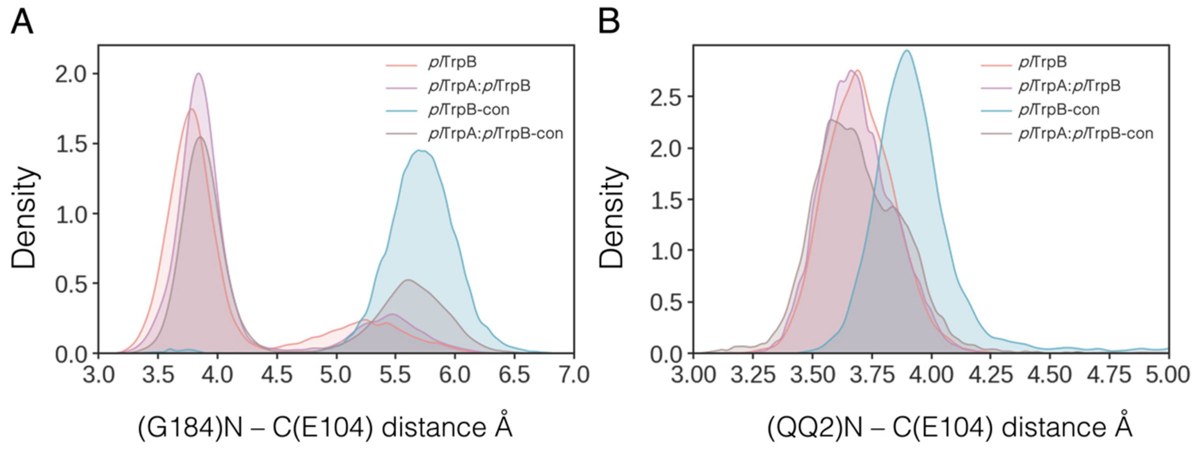

Figure S12. Histogram of the stabilization distance between (**A**) G184 backbone nitrogen and E104 delta carbon, and (**B**) QQ2 nitrogen and E104 delta carbon (in Å) for: *pl*TrpB (in orange), *pl*TrpA:*pl*TrpB (in purple), *pl*TrpB-con (in teal) and *pl*TrpA:*pl*TrpB-con (in brown).

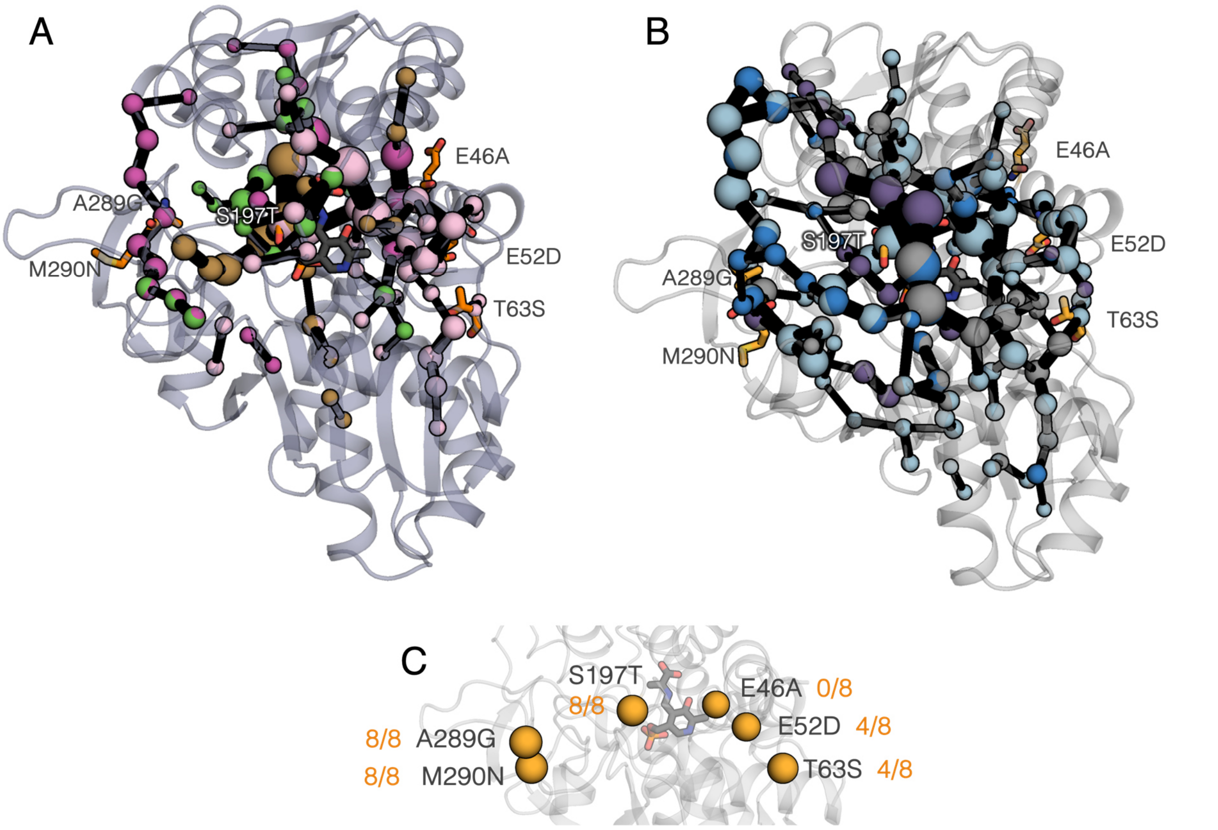

Figure S13. SPM pathways considering the AA intermediate within *pl*TrpB (magenta), *plTrpA*:*pl*TrpB (pink), *pl*TrpB-con (green) and *pl*TrpA:*pl*TrpB-con (brown) in panel (**A**), and *pf*TrpB (blue), 0B2-*pf*TrpB [4, 5] (purple), LBCA-TrpB [6] (light blue) and SPM6-TrpB[6] (gray) in panel (**B**). The occurrence of Res_6 positions in the networks analyzed from the eight different systems (coming from different organisms) is shown in panel (**C**).

The results indicate that the active site substitution S197 plays a central role in the whole conformational network, positions A289, M290 act synergistically and potentially impact the conformation of H275 involved in the opening/closure of the TrpA-TrpB tunnel region. Positions E52 and T63 are identified in half (4/8) of the constructed SPMs despite being less connected to the central network. Finally, E46 is not included in any SPM (0/8) but is adjacent to SPM positions in 2/8 cases, thus suggesting a lower contribution of this position. This analysis based on SPM conformational networks supports the idea of a general effect of these Res_6 positions regardless of the scaffold context. The combination of SPM and MSA indicates that the six positions present different level of conservation, being T63 and S197 more conserved, followed by A289, whereas M290, E46 and E52 displaying a larger dispersion of amino acids.

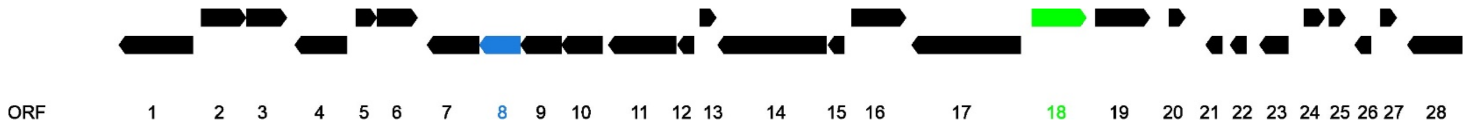

ORF	ORIENTATION	FUNCTIONAL ANNOTATION
**1**	-	Glycosyl transferase
**2**	+	Methionine adenosyltransferase
**3**	+	Cation efflux protein
**4**	-	K+-dependent Na+/Ca+ exchanger related-protein
**5**	+	Coenzyme Q-binding protein COQ10 START domain-containing protein
**6**	+	Nucleoside-diphosphate-sugar epimerases-like protein
**7**	-	Glycosyl transferase
**8**	-	Tryptophan synthase alpha chain
**9**	-	Abortive infection protein
**10**	-	7-cyano-7-deazaguanine synthase
**11**	-	Chaperonin GroEL
**12**	-	Co-chaperonin GroES
**13**	+	Uncharacterized protein
**14**	-	Excinuclease ABC subunit A
**15**	-	Transcriptional regulator
**16**	+	Dihydrolipoamide acetyltransferase
**17**	-	LPS-assembly protein LptD central domain-containing protein
**18**	+	Tryptophan synthase beta chain
**19**	+	Amino acid ABC transporter substrate-binding protein
**20**	+	Rhodanese-like protein
**21**	-	Uncharacterized protein
**22**	-	DUF3127 domain-containing protein
**23**	-	VWA domain-containing protein
**24**	+	Transcriptional regulator
**25**	+	Exonuclease
**26**	-	Leucine-rich repeat domain-containing protein
**27**	+	Chlorosome envelope protein B
**28**	-	Pep581 peptidase

Figure S14. Genome neighborhood of *pltrpA* and *pltrpB*. Analysis of the genomic neighborhood reveals several striking differences compared to other TrpB1 enzymes: First, *pltrpA* (ORF 8) and *pltrpB* (ORF 18) are separated by 9 intervening genes. Second, *pltrpA* (-) and *pltrpB* (+) are encoded on different DNA strands, thus not co-transcribed. Neither *pltrpA* nor *pltrpB* is embedded in a *trp* operon.

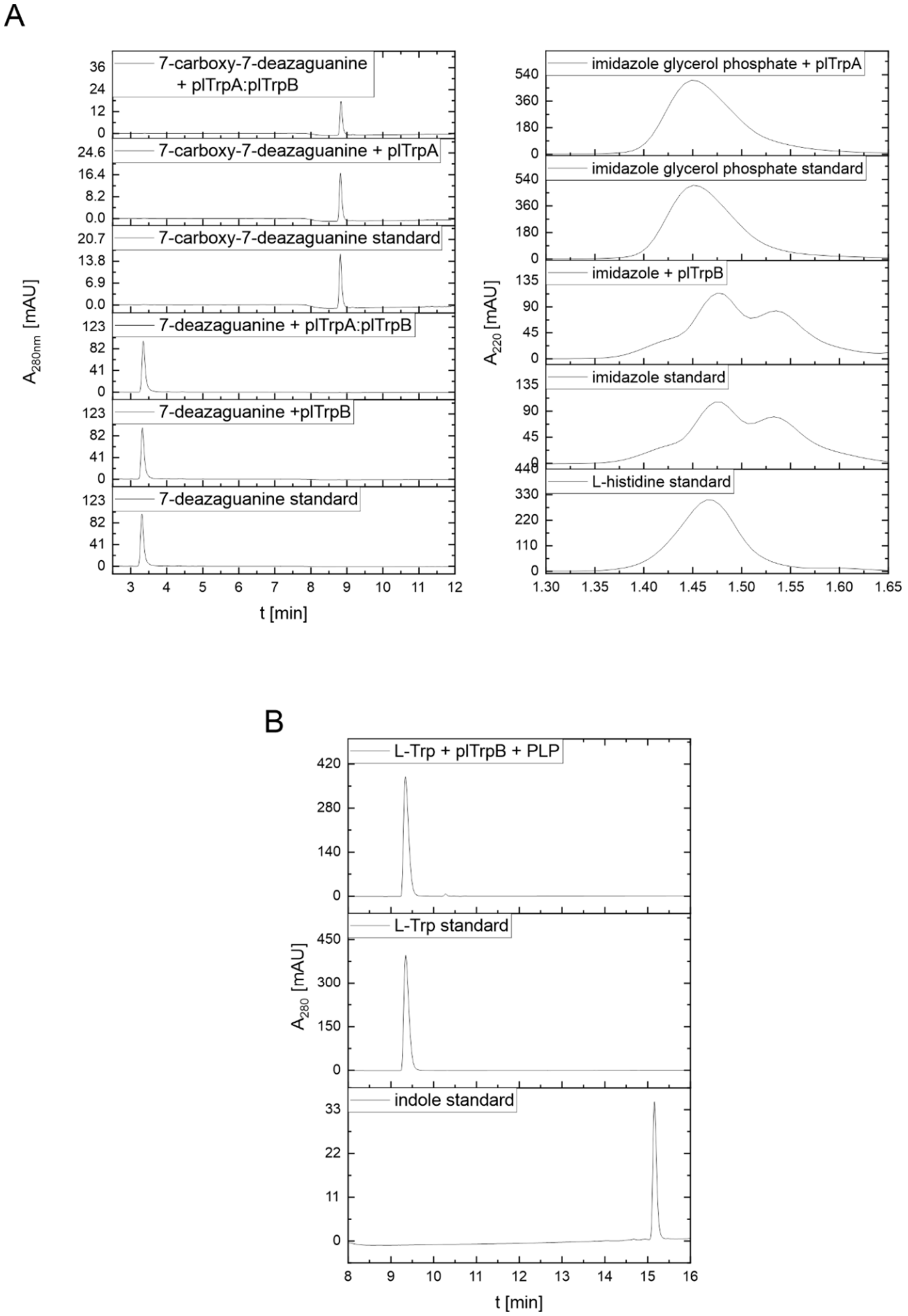

Figure S15. HPLC-based screening with potential alternative substrates of *pl*TrpB, *pl*TrpA, and *pl*TrpA:*pl*TrpB. (**A**) All enzymatic assays contained 500 µM of the tested substrate, 100 mM potassium phosphate (pH 7.5), 180 mM KCl, 40 µM PLP, 10 µM enzyme, and 2 mM L-serine. (**B**) The reverse reaction was tested using 2mM L-Trp, 100 mM potassium phosphate (pH 7.5), 180 mM KCl, 40 µM PLP, and 10 µM *pl*TrpB. All samples were incubated for 60 min at 30 °C while shaking at 500 rpm on an Eppendorf table top shaker.

Table S1. Assignment of *pl*TrpB to the TrpB1 group.

	Characteristic active site residue	Main alpha-amino acid substrate	Sequence identity to (other) TrpB1 enzymes	Sequence identity to (other) TrpB2 enzymes
**TrpB1 enzymes**	D	L-serine	~ 60%	~ 30%
**TrpB2 enzymes**	R	O-phospho-L-serine	~ 30%	~ 60%
***pl*TrpB**	D	L-serine	~ 60%	~ 30%

Table S2. Characteristics and amino acid sequences of *pl*TrpA, *pl*TrpB, and *pl*TrpB-con.

Protein	MW [Da]	ε_{280} [$M^{-1}cm^{-1}$]	Number of amino acids	Sequence
***Pelodictyon luteolum* TrpA (*pl*TrpA)**	30461	26930	278	MTQKKENRITARLREDRKLLLAYYPEFP<br>VAGSTLPVLEALQDGGADIIELGIPFSDP<br>VGDGPVIQNAAHIAIRNGVSVRSLLELV<br>RKARAGEGCRKITVPILLMGYSNPLIAY<br>GGDCFLHDAVKAGVDGLLIPDLPPEES<br>ADFLQRAKSLGLTVVYLISPVTPPERIE<br>WIDSLSTDFSYCLAVNATTGTAKLADAS<br>TEASVDRYLERVRLHARKKFVVGFGIR<br>DRARVEHMWRLADGAVVGTALLEHIAG<br>APNPGEAARRAGEFWRGLRLEHHHHH<br>H
***Pelodictyon luteolum* TrpB (*pl*TrpB)**	43880	27390	407	MSTTAYSAPDSAGKFGAFGGRFTPETL<br>MQNAAGLEAEYLKAKSDPEFQRTLEGL<br>LGDYVGRPTPLYHAEGLSGMLGGAEV<br>WLKREDLCHTGAHKINNALGQVLLARR<br>MGKRRVIAETGAGQHGVATATVCALFN<br>LQCVVYMGEEDIRRQAPNVARMKLLGA<br>EVRPVSSGSRTLKDATSEAIRDWMNNP<br>EETFYIIGSVVGMHPYPMIVRDFQAVIG<br>RETRRQILDKAGRLPDVITACIGGGSNA<br>VGMFYDFLPDAGSIELIGVEAAGEGLDR<br>RHAASLTMGSPGVLHGAMTMLLQNED<br>GQIQEAHSISAGLDYPGVGPEHCHLQE<br>LGLVKYTSVTDADALSALRTLAETEGIIC<br>ALESAHAVRYAITRAPEMAKDKIIIVNLS<br>GRGDKDMGTIMEELNLLEHHHHHH
***Pelodictyon luteolum* TrpB-con (*pl*TrpB-con)**	43777	27390	407	MSTTAYSAPDSAGKFGAFGGRFTPETL<br>MQNAAGLEAEYLKAKSDPAFQRTLDGL<br>LGDYVGRPSPLYHAEGLSGMLGGAEV<br>WLKREDLCHTGAHKINNALGQVLLARR<br>MGKRRVIAETGAGQHGVATATVCALFN<br>LQCVVYMGEEDIRRQAPNVARMKLLGA<br>EVRPVSSGSRTLKDATSEAIRDWMNNP<br>EETFYIIGTVVGMHPYPMIVRDFQAVIG<br>RETRRQILDKAGRLPDVITACIGGGSNA<br>VGMFYDFLPDAGSIELIGVEAAGEGLDR<br>RHAASLTMGSPGVLHGGNTMLLQNED<br>GQIQEAHSISAGLDYPGVGPEHCHLQE<br>LGLVKYTSVTDADALSALRTLAETEGIIC<br>ALESAHAVRYAITRAPEMAKDKIIIVNLS<br>GRGDKDMGTIMEELNLLEHHHHHH

Calculation S1 - Estimation of the TrpA:TrpB complex concentration.

Activity titrations of TrpB with TrpA yielded apparent K_d^{app} values of 8.5 μM for *pl*TrpA:*pl*TrpB and 9.9 μM for *pl*TrpA:*pl*TrpB-con. This means that for full complex formation concentrations well above 10 μM would have been needed in the kinetic experiments. However, such high protein concentrations would have greatly increased the basal absorption at 290 nm, the wavelength which was also used to monitor the progress of the reaction, effectively leading to absorptions in a range where Lambert-Beer's law is no longer applicable, thus hampering data acquisition. To account for the high K_d values, we therefore opted to calculate the fraction of TrpA:TrpB complex under the assay conditions used for the steady-state kinetic experiments assuming identical K_d values for both the formation of the αββ trimer and the αββα tetramer (cf. **Figure S9**).

A_t = concentration of total TrpA

B_t = concentration of total TrpB

AB = concentration of TrpA:TrpB complex

A = concentration of free TrpA = A_t - AB

B = concentration of free TrpB = B_t - AB.

K_d is then defined as:

$$K_d = \frac{A * B}{AB}$$

$$K_d = \frac{(A_t - AB) * (B_t - AB)}{AB}$$

$$K_d * AB = A_t * B_t - A_t * AB - B_t * AB + AB^2$$

$$0 = AB^2 - (A_t + B_t + K_D) * AB + A_t * B_t$$

Solving the equation for the concentration of TrpA:TrpB yields:

$$AB = \frac{(A + B + K_d) \pm \sqrt{(A + B + K_d)^2 - 4 * 1 * A_t * B_t}}{2}$$

For the kinetic experiments in the complex, 3 μM TrpB and a 1.5-fold excess of TrpA, i.e., 4.5 μM TrpA were used. The K_d for *pl*TrpA:*pl*TrpB was 8.5 μM while the K_d for *pl*TrpA:*pl*TrpB-con was 9.9 μM.

For *pl*TrpA:*pl*TrpB follows:

$$AB = \frac{(4.5 + 3 + 8.5) - \sqrt{(4.5 + 3 + 8.5)^2 - 4 * 4.5 * 3}}{2} \mu M \approx 0.89\ \mu M$$

For *pl*TrpA:*pl*TrpB-con follows:

$$AB = \frac{(4.5 + 3 + 9.9) - \sqrt{(4.5 + 3 + 9.9)^2 - 4 * 4.5 * 3}}{2} \mu M \approx 0.81\ \mu M$$

As a consequence, 30% (0.89/3) and 27% (0.81/3) of the TrpB molecules were in complex with TrpA for *pl*TrpA:*pl*TrpB and *pl*TrpA:*pl*TrpB-con measurements, respectively. The remaining 70 % for *pl*TrpA:*pl*TrpB and 73% for *pl*TrpA:*pl*TrpB-con were not complexed with TrpA.

Therefore, the k_{cat} values of the complexes were underestimated in both cases. They can be calculated as follows:

$k_{cat,obs}$ = k_{cat} value of the complex observed under the experimental conditions in the kinetic experiments

$k_{cat,mon}$ = k_{cat} value of TrpB in absence of TrpA

$k_{cat,com}$ = k_{cat} value of the TrpA:TrpB complex

f_B = fraction of TrpB which is not complexed with TrpA

f_{AB} = fraction of TrpB in complex with TrpA.

$$k_{cat,obs} = k_{cat,mon} * f_B + k_{cat,com} * f_{AB}$$

$$k_{cat,com} = \frac{k_{cat,obs} - k_{cat,mon} * f_B}{f_{AB}}$$

	$k_{cat,obs}$ [s^{-1}]	$k_{cat,mon}$ [s^{-1}]	f_B	f_{AB}	$k_{cat,com}$ [s^{-1}]	$k_{cat,com}$/ $k_{cat,obs}$	$k_{cat,com}$/ $k_{cat,mon}$
*pI*TrpA:*pI*TrpB	0.93	0.35	0.7	0.3	2.28	2.5	6.5
*pI*TrpA:*pI*TrpB-con	0.21	0.009	0.73	0.27	0.75	3.6	84

This means that the actual k_{cat} values of TrpB in the complex might be 2.5-fold higher for *pI*TrpA:*pI*TrpB and 3.6-fold higher for *pI*TrpA:*pI*TrpB-con than what was measured in the kinetic experiments. The activation upon complex formation therefore would be 6.5-fold and 84-fold.

References

1. Raboni S, Bettati S, Mozzarelli A. Tryptophan synthase: a mine for enzymologists. *Cell Mol Life Sci*. 2009; **66**: 2391–2403.
2. Wilkins MR, Gasteiger E, Bairoch A, Sanchez JC, Williams KL et al. Protein identification and analysis tools in the ExPASy server. *Methods Mol Biol*. 1999; **112**: 531–552.
3. Buller AR, Brinkmann-Chen S, Romney DK, Herger M, Murciano-Calles J et al. Directed evolution of the tryptophan synthase β-subunit for stand-alone function recapitulates allosteric activation. *Proc Natl Acad Sci U S A*. 2015; **112**: 14599–14604.
4. Calvó-Tusell C, Maria-Solano MA, Osuna S, Feixas F. Time Evolution of the Millisecond Allosteric Activation of Imidazole Glycerol Phosphate Synthase. *J Am Chem Soc*. 2022; **144**: 7146–7159.
5. Duran C, Casadevall G, Osuna S. Harnessing conformational dynamics in enzyme catalysis to achieve nature-like catalytic efficiencies: the shortest path map tool for computational enzyme redesign. *Faraday Discuss*. 2024; **252**: 306–322.
6. Maria-Solano MA, Kinateder T, Iglesias-Fernández J, Sterner R, Osuna S. In Silico Identification and Experimental Validation of Distal Activity-Enhancing Mutations in Tryptophan Synthase. *ACS Catal*. 2021; **11**: 13733–13743.
